# Yin Yang 1 promotes aggressive cell growth in high‐grade breast cancer by directly transactivating kinectin 1

**DOI:** 10.1002/mco2.133

**Published:** 2022-07-05

**Authors:** Lin Gao, Wenbin Zhou, Ni Xie, Junying Qiu, Jingyi Huang, Zhe Zhang, Malin Hong, Jinquan Xia, Jing Xu, Pan Zhao, Li Fu, Yuwei Luo, Jing Jiang, Hui Gong, Jigang Wang, Yong Dai, Dixian Luo, Chang Zou

**Affiliations:** ^1^ Department of Clinical Medical Research Center The Second Clinical Medical College Jinan University (Shenzhen People's Hospital) The First Affiliated Hospital of Southern University of Science and Technology Shenzhen Guangdong China; ^2^ Department of Thyroid and Breast Surgery Department of General Surgery The Second Clinical Medical College Jinan University (Shenzhen People's Hospital) The First Affiliated Hospital of Southern University of Science and Technology Shenzhen Guangdong China; ^3^ Biobank Shenzhen Second People’ s Hospital Shenzhen, Health Science Center First Affiliated Hospital of Shenzhen University Shenzhen Guangdong China; ^4^ Medical Laboratory of Shenzhen Luohu People's Hospital Shenzhen Guangdong China; ^5^ Shenzhen Public Service Platform on Tumor Precision Medicine and Molecular Diagnosis the Second Clinical Medical College Jinan University Shenzhen Guangdong PR China; ^6^ Guangdong Provincial Key Laboratory of Regional Immunity and Diseases Department of Pharmacology and International Cancer Center Shenzhen University Health Science Center Shenzhen Guangdong China; ^7^ Department of Laboratory Medicine Huazhong University of Science and Technology Union Shenzhen Hospital (Nanshan Hospital) Shenzhen Guangdong China; ^8^ School of Life and Health Sciences The Chinese University of Kong Hong Shenzhen Guangdong China

**Keywords:** breast cancer, DDX3X, growth, invasion, KTN1, YY1

## Abstract

Invasive cancer growth and metastasis account for the poor prognosis of high‐grade breast cancer. Recently, we reported that kinectin 1 (KTN1), a member of the kinesin‐binding protein family, promotes cell invasion of triple‐negative breast cancer and high‐grade breast cancer cells by augmenting the NF‐κB signaling pathway. However, the upstream mechanism regulating KTN1 is unknown. Therefore, this functional study was performed to decipher the regulatory cohort of KTN1 in high‐grade breast cancer. Bioinformatic analysis indicated that transcription factor Yin Yang 1 (YY1) was a potential transactivator of KTN1. High YY1 expression correlated positively with pathological progression and poor prognosis of high‐grade breast cancer. Additionally, YY1 promoted cell invasive growth both in vitro and in vivo, in a KTN1‐dependent manner. Mechanistically, YY1 could transactivate the KTN1 gene promoter. Alternatively, YY1 could directly interact with a co‐factor, DEAD‐box helicase 3 X‐linked (DDX3X), which significantly co‐activated YY1‐mediated transcriptional expression of KTN1. Moreover, DDX3X augmented YY1‐KTN1 signaling‐promoted invasive cell growth of breast cancer. Importantly, overexpression of YY1 enhanced tumor aggressive growth in a mouse breast cancer model. Our findings established a novel DDX3X‐assisted YY1‐KTN1 regulatory axis in breast cancer progression, which could lead to the development novel therapeutic targets for breast cancer.

## Graphical abstract

Invasive cancer cell growth and metastasis accounts for the poor prognosis of high‐grade breast cancer. The transcription factor Yin Yang 1 (YY1) is upregulated in high‐grade breast cancer. YY1 interacted with DEAD‐box protein (DDX3X) via binding to DDX3X. YY1 transactivated *KTN1* gene via direct binding its promoter together with the DDX3X. Herein, our findings established a novel YY1‐assisted DDX3X‐*KTN1* regulatory axis, which would shed some light on the signaling involved in cell invasion and progression in breast cancer and open an avenue in finding novel therapeutic targets for breast cancer.

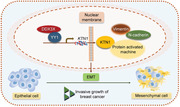

## 1 INTRODUCTION

Despite of the marked improvement in the targeted therapy in recent decades, breast cancer (BCa) remains an incurable disease in its advanced stage.^1^ Pathologically, BCa grade depends on the degree of differentiation of cancer tissues, and invasive tumor growth and metastasis accounts for the progression and relapse of most BCa.^2^ Recent studies have uncovered multiple genes and signaling pathways that regulate the progression of BCa.^3^, ^4^

Kinectin (KTN1) is a kinesin‐binding protein that can modulate the reorganization of micro‐tubules and intracellular organelle transport.^5^, ^6^ Studies have demonstrated that elevated KTN1 expression correlates positively with disease progression in many cancer types, such as bladder cancer, cutaneous squamous cell carcinoma, and BCa.^7^, ^8^, ^9^ Moreover, *KTN1* overexpression could promote cell proliferation and migration in cutaneous squamous cancer cells, indicating a functional role of KTN1 in invasive tumor growth. Our previous findings also confirmed that KTN1 can phosphorylate NF‐kappa B (NF‐κB) p65 subunit by combining specifically with p65, and the complex accelerated BCa growth via transactivating C–X–C motif chemokine ligand 8 (CXCL8).^9^ Additionally, high KTN1 expression correlated positively with the expression levels of mesenchymal biomarkers, whereas it inhibited the expression of epithelial biomarkers in BCa. However, the molecular mechanism needs to be further determined.

Yin Yang 1 (YY1), a zinc finger protein, is a member of the GLI‐Kruppel family.^10^ As a transcription factor, its downstream target genes are involved in series of the cellular process in tumor progression, including cell proliferation, invasion, metastasis, and angiogenesis.^11^ Structurally, YY1 has an activation or inhibition domain, which is in N‐terminus or C‐terminus, respectively.^11^ Therefore, YY1 can activate or repress the transcription of its downstream genes depending on the binding of its interacting co‐factor to the promoters of these targeted genes.^12^ Increasing evidence suggests that YY1 can promote the development and progression of many cancers.^13^ However, its functional role in BCa progression is controversial.^14^ YY1 can promote Erb‐B2 receptor tyrosine kinase 2 (ERBB2) subtype of BCa invasion by upregulating the expression of *ERBB2* in co‐operation with its transcriptional co‐activator activator protein 2 (AP‐2).^15^, ^16^ Alternatively, YY1 can suppress cell proliferation via breast cancer type 1 susceptibility protein (*BRCA1)* expression in BCa. YY1 can bind to and regulate the *BRCA1* promoter positively.^11^ However, the exact function of YY1 in high‐grade BCa, especially in the invasive growth of BCa, needs to be clarified.

*DDX3X* (encoding DEAD‐box helicase 3X‐linked, also known as DDX3X, DDX3, DBX) belongs to the DEAD‐box helicase gene family. Accumulating evidence indicates that DDX3X plays an essential role in embryonic development and cancer progression, modulating multiple biological processes, such as gene transcription, pre‐mRNA splicing, and protein translation.^17^ Recent studies revealed that upregulated expression of *DDX3X* in BCa could promote tumorigenesis and cell proliferation by accelerating the cell cycle.^18^ Moreover, depletion of *DDX3X* in BCa cells inhibited its lung metastasis.^19^ These findings suggested a pro‐oncogenic role of DDX3X in BCa progression.^20^

In our study, we aimed to determine whether YY1 is a key transcription factor and positive regulator of *KTN1* transcription. Upregulated YY1 expression was linked strongly to poor outcome of high‐grade BCa. We also identified that DDX3X could augment YY1‐*KTN1* signaling‐mediated BCa invasive growth by interacting with YY1 and co‐activating YY1‐induced transcription of *KTN1* in BCa cells. These findings clarified a pro‐oncogenic role of YY1 in high‐grade BCa, in which it binds to DDX3X to promote the aggressive growth of BCa by activating *KTN1*. Our findings could facilitate unraveling the molecular basis of BCa progression and the development novel treatment strategies to overcome this disease.

## 2 RESULTS

### 2.1 YY1 is a potential upstream transcription factor of *KTN1* in high‐grade BCa

**FIGURE 1:**
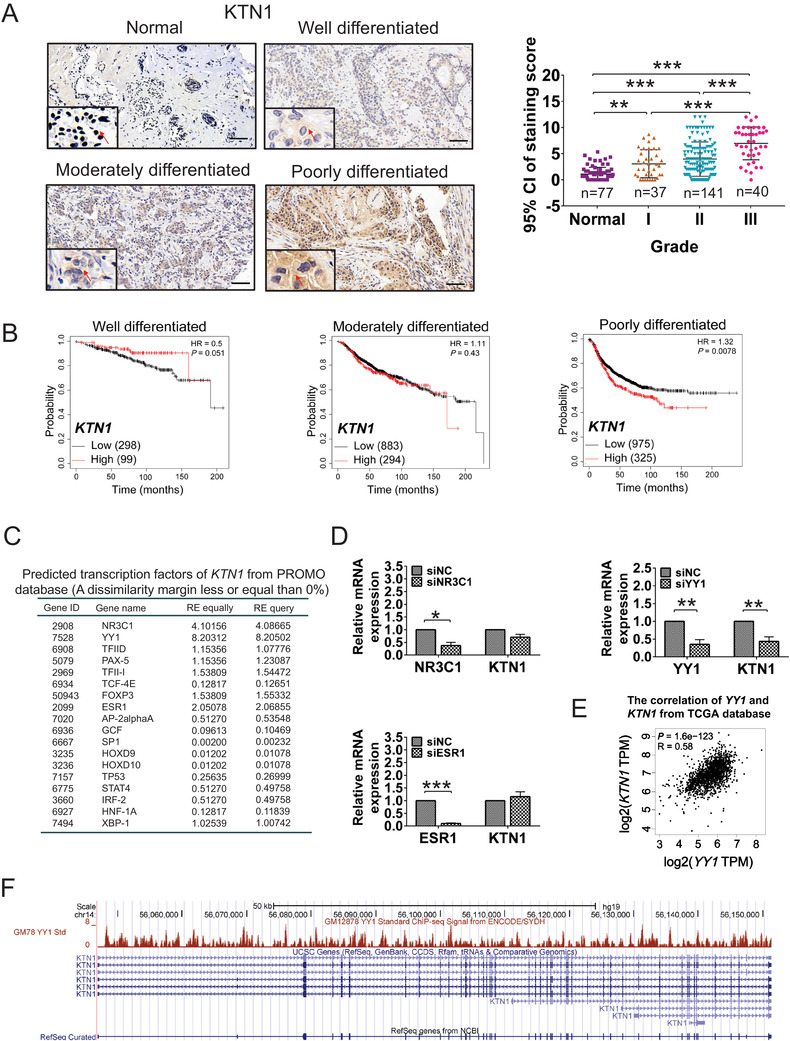
YY1 was an underlying transcription factor that regulates *KTN1* expression in high‐grade breast cancer (BCa). (A) Immunohistochemistry (IHC) staining of KTN1 in BCa tissues. Grade I (a total score of 3–5, well differentiated), Grade II (a total score of 6–7, moderately differentiated), and Grade III (a total score of 8–9, poorly differentiated). Positive signals (brown staining) of KTN1 are indicated by arrow heads. The number of cases is indicated below. All data were plotted as the means of the 95% confidence interval plus the s.d. (B) Kaplan–Meier analysis of well differentiated, moderately differentiated, and poorly differentiated tumors for relapse‐free survival (RFS) with high versus low expression levels of *KTN1* mRNA. (C) Bioinformatic analysis of predicted upstream transcription factors of *KTN1* from the PROMO database. (D), *NR3C1*, *ESR1*, *YY1*, and *KTN1* expression was identified by qRT‐PCR in MDA‐MB‐231 cells treated with siRNA oligonucleotides and negative control group (siNC) oligonucleotides, respectively. (E) The correlation analysis between *YY1* and *KTN1* expression from the GEPIA database (*R* = 0.58, *P *= 1.6e−123). (F) Coverage plot analysis of the transcription start site and promoter site of the *KTN1* gene based on Chromatin Immunoprecipitation‐sequencing (ChIP‐seq) assay from Richard Myers data from the UCSC database. Error bars are shown with the s.d., *n* ≥ 3. * *P *< 0.05, ** *P *< 0.01 and *** *P *< 0.001 compared with the negative control groups. A two‐tailed *t*‐test or ANOVA was used to assess the *P*‐values. Scale bars, 100 μm

A previous study suggested that upregulation of KTN1 promoted BCa malignancy.^9^ To explore the upstream regulatory mechanism of *KTN1* regulation, the expression levels of KTN1 were analyzed in different pathologically grades of BCa using immunohistochemistry (IHC) staining [Grade I (a total score of 3–5, well differentiated), Grade II (a total score of 6–7, moderately differentiated), and Grade III (a total score of 8–9, poorly differentiated)]. The results showed that KTN1 protein levels were high in Grade I‐III BCa tissues compared with that in adjacent normal tissues, particular among Grade III samples. Furthermore, KTN1 protein staining was confirmed to increase gradually with histological grade (Figure 1A).

To reveal the interrelation between KTN1 and histological grades in clinical prognosis of BCa, we performed the outcomes of BCa patients through the Kaplan–Meier plotter online. The results identified that upregulated expression of *KTN1* mRNA was associated with poor clinical prognosis accompanied by progression of BCa (Grade I, *P *= 0.051; Grade II, *P *= 0.43; Grade III, *P *= 0.0078, Figure 1B). These findings indicated that KTN1 might be a prognostic biomarker for high‐grade BCa and promotes BCa tumorigenesis.

To investigate the upstream transcription factors that modulate the *KTN1* gene, we predicted transcription factors that might bind to the *KTN1* promoter by the PROMO database (http://algge n.lsi.upc.es/cgi‐bin/promo_v3/promo/promoinit.cgi?dirDB = TF8.3) to screen for known transcription factor binding sites.^21^ The score from random expectation (RE) analysis identified the top ranked genes as *NR3C1*, *YY1*, and *ESR1* (conforming to RE equally ≥ 2 & RE query ≥ 2, Figure 1C). Next, quantitative real‐time reverse transcription (qRT‐PCR) analysis showed that knockdown of *NR3C1* using *NR3C1* siRNA oligonucleotides (siNR3C1) had no significant effect on the regulation *KTN1* expression compared with the negative control group (siNC). Primer sequences are listed in Table S3. Similar results were observed in the cell line with *ESR1* knockdown using siESR1 oligonucleotides. By contrast, *YY1* knockdown led to markedly reduced *KTN1* expression (Figure 1D). We also performed that the mRNA expression of *YY1* correlated positively with the mRNA expression of *KTN1* based on GEPIA data (http://gepia.cancer‐pku.cn/, *R* = 0.58, *P *= 1.6e−123, Figure 1E).^22^ Non‐significant correlations between *NR3C1* or *ESR1* and *KTN1* were observed (Figure S1A).

Furthermore, we analyzed approximately 2000 bp of the *KTN1* promoter binding region by the UCSC genome browser (http://genome.ucsc.edu/) [Data obtained from Richard Myers data (GEO:GSM803535, UCSC‐ENCODE‐hg19: wgEncodeEH001573)], which was found to recruit YY1 at multiple binding sites (Figure 1F). These results revealed that YY1 might be a pivotal regulator of *KTN1* expression in high‐grade BCa.

### 2.2 Elevated expression of YY1 correlates positively with poorly clinical outcomes in high‐grade BCa

**FIGURE 2:**
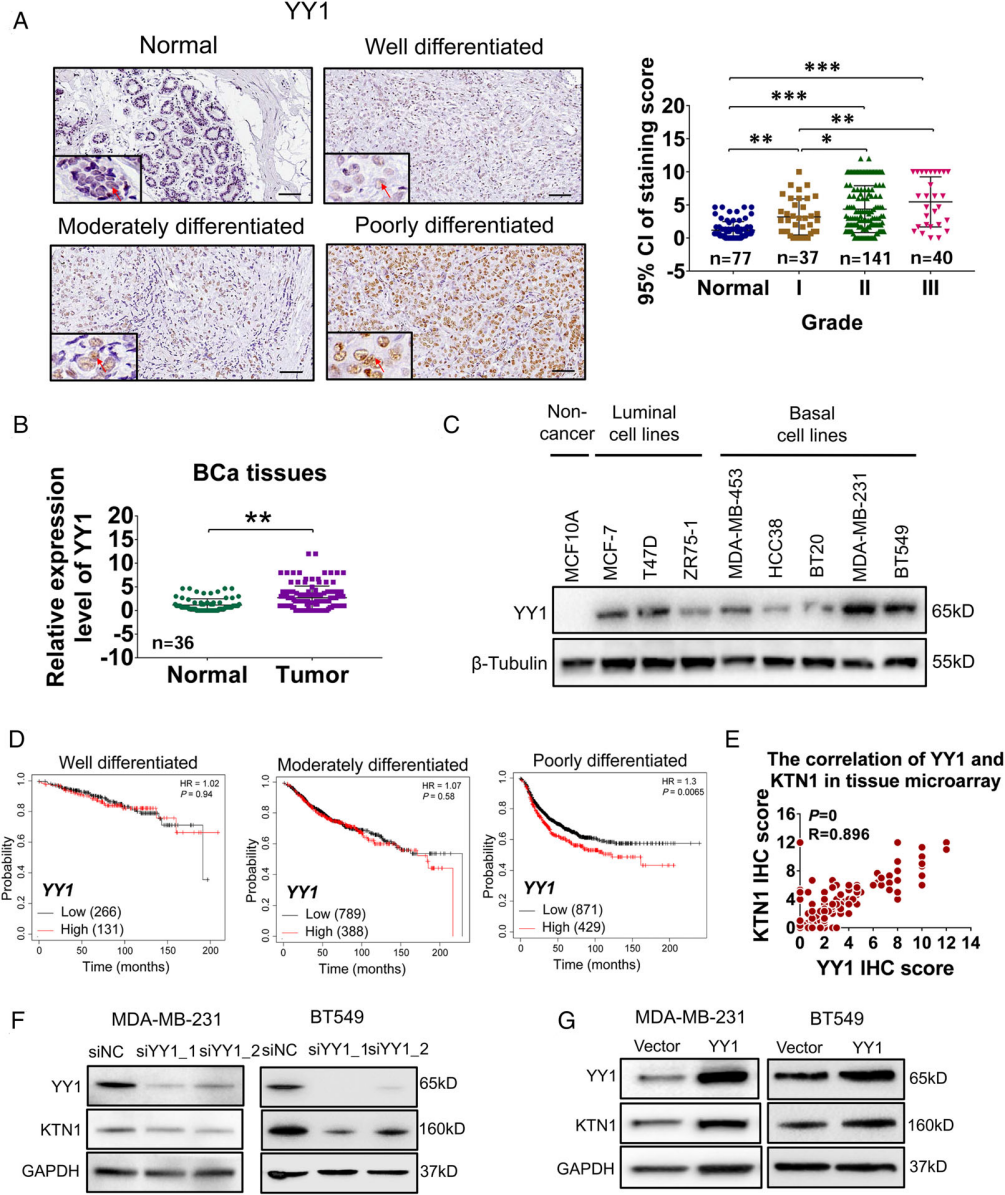
High YY1 expression was associated with poor clinical prognosis. (A) Immunohistochemistry (IHC) staining of YY1 in breast cancer (BCa) tissues. All data are shown as the means of the 95% confidence interval plus the s.d. (B) The expression level of *YY1* mRNA was examined by qRT‐PCR in 36‐paired BCa tissues compared to adjacent normal tissues. (C) Western blotting analysis of the level YY1 protein in MCF10A, MCF‐7, T47D, ZR75‐1, MDA‐MB‐453, HCC38, BT20, MDA‐MB‐231, and BT549 cell lines. (D) Kaplan–Meier analysis of well differentiated, moderately differentiated, and poorly differentiated for relapse‐free survival (RFS) with high versus low expression levels of *YY1* mRNA from the GEPIA database. (E) Correlation analysis between YY1 and KTN1 from the tissue microarray (*R* = 0.896, *P *= 0). (F) Knockdown of *YY1* in MDA‐MB‐231 and BT549 cell lines treated with negative control (siNC), YY1_1 and YY1_2 siRNA oligonucleotides was determined using western blotting assays. (G) Overexpression of *YY1* in both BCa cell lines transfected with empty vector or YY1 overexpression plasmid was detected using western blotting analysis. The *P*‐value of data showed significant differences as indicated using * *P *< 0.05, ** *P *< 0.01 and *** *P *< 0.001

The transcription factor YY1 is elevated in various types of cancers and promotes tumor growth and metastasis.^23^ However, the regulatory mechanism of YY1 in high‐grade BCa is poorly understood. An IHC assay using a tissue microarray showed that YY1 was increased in BCa tissues compared to that in adjacent normal tissues. YY1 protein levels were higher in poorly differentiated BCa tissues compared to those in well differentiated BCa tissues (Figure 2A). Additionally, qRT‐PCR analysis was confirmed that the expression of *YY1* was increased observably in BCa tissues compared with that in paracancerous tissues (*n* = 36) (Figure 2B). Analogous results were obtained from BCa cell lines using western blotting. As shown in Figure 2C, the level of YY1 was higher in BCa cell lines, especially basal cells, MDA‐MB‐231 and BT549, compared with that in the human mammary epithelial cell line MCF10A. A previous study showed that patients with triple negative BCa exhibited higher histological grade and worse prognosis than other subtypes of BCa.^24^ The correlation between YY1 expression and clinical outcomes was analyzed according to Kaplan–Meier plotter dataset online. The results showed that high YY1 expression correlated positively with decreased RFS along with BCa malignancy (well differentiated, *P *= 0.94; moderately differentiated, *P *= 0.56; poorly differentiated, *P *= 0.0065, respectively, Figure 2D). However, high ESR1 and NR3C1 levels were not associated with poor outcome of high‐grade BCa (Figure S1B). Thus, these findings suggested that YY1 acts as an oncogene, and is highly expressed and correlated with poor outcomes in high‐grade BCa.

Next, a similar positive correlation of YY1 protein levels was observed in the tissue microarray data (*R* = 0.896, *P *= 0, Figure 2E). Moreover, to verify whether YY1 regulates the expression of *KTN1*, *YY1* was knocked down using siRNA oligonucleotides in both cell lines (Figure 2F, Figure S2A). The results showed that the expression of the KTN1 decreased markedly in cells treated with siYY1 compared to that siNC group. By contrast, overexpression of *YY1* increased the level of the KTN1 protein in both BCa cell lines (Figure 2G, Figure S3A). Taken together, these findings suggested that upregulated YY1 expression resulted in poor clinical prognosis of high‐grade BCa by regulating *KTN1* expression.

### 2.3 YY1 promotes the growth and epithelial–mesenchymal transition of BCa cells in a KTN1‐dependent manner in vitro and in vivo

**FIGURE 3:**
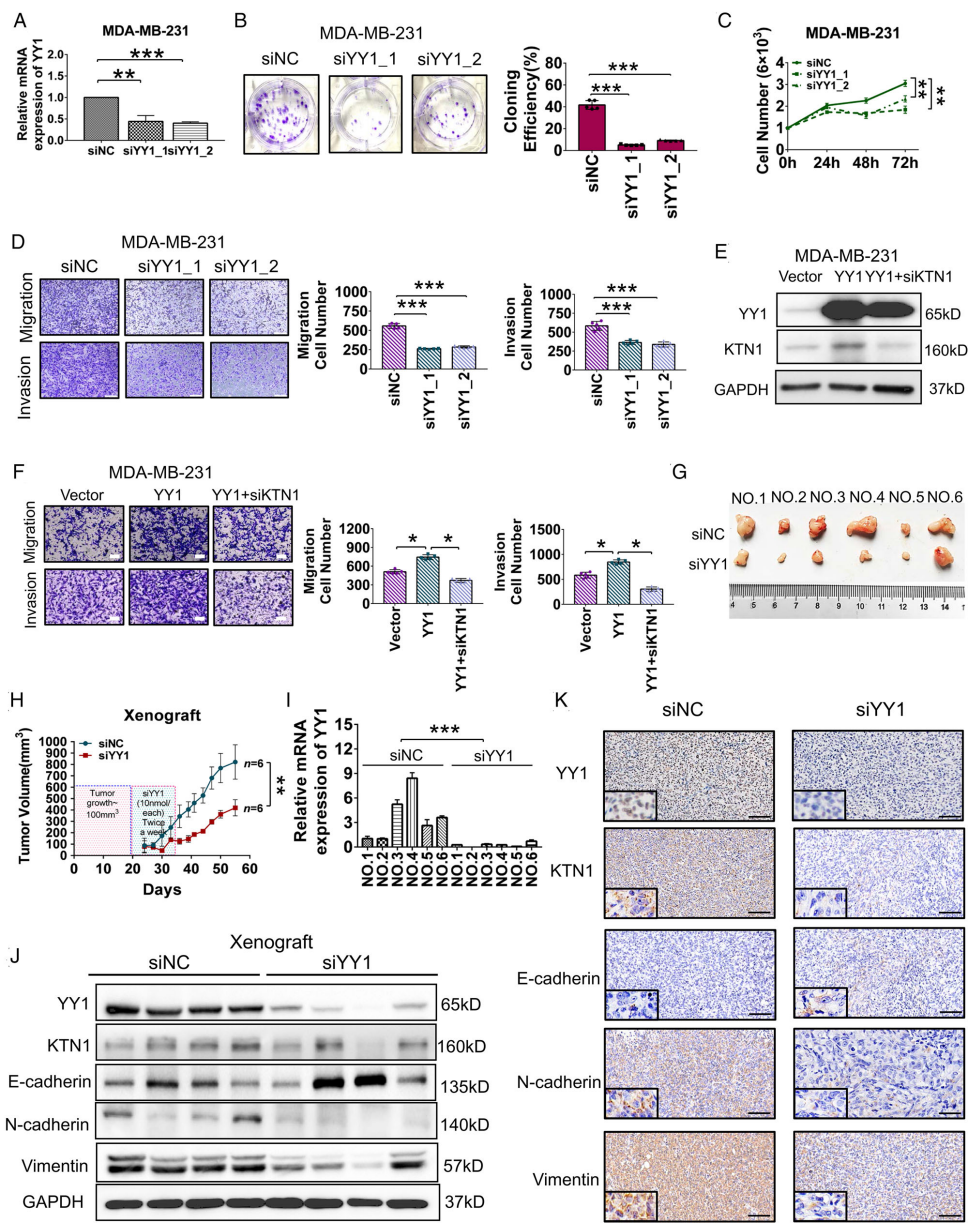
Depletion of YY1 blocked the invasive growth of breast cancer (BCa) cells in vitro and in vivo. (A) Knockdown of *YY1* in MDA‐MB‐231 cells treated with siYY1_1 or siYY1_2 oligonucleotides compared with the siNC group as assessed by a qRT‐PCR assay. (B) CCK‐8 assay showing the proliferation of MDA‐MB‐231 cells treated with siNC and siYY1 oligonucleotides. (C) Monolayer colony formation assay showing the colony forming efficiencies of MDA‐MB‐231 cells treated with siNC and siYY1 oligonucleotides. (D) Transwell assay showing the migration and invasion of MDA‐MB‐231 cells treated with siNC and siYY1 oligonucleotides. (E) Western blotting analysis of protein levels in *YY1*‐overexpressing MDA‐MB‐231 cells treated with siKTN1 oligonucleotides. (F) Migration and invasion analysis in ‐overexpressing MDA‐MB‐231 cells treated with siKTN1 oligonucleotides using a Transwell assay. (G) Knockdown of *YY1* with siYY1 oligonucleotides attenuated MDA‐MB‐231 cell growth in a mouse xenograft model compared with that in the siNC group. (H) Tumor volumes were measured after injection of MDA‐MB‐231 cells with siYY1 oligonucleotides in the xenograft mouse model; *n* = 6. (I) The expression levels of *YY1* mRNA in the xenograft tumors. (J) Western blotting assay to detect the protein levels of YY1, KTN1, and epithelial‐to‐mesenchymal transition (EMT) markers. (K) Immunohistochemistry (IHC) staining of YY1, KTN1, and EMT marker in xenograft tumors. Error bars are shown with the s.d., *n* ≥ 3. * *P *< 0.05, ** *P *< 0.01 and *** *P *< 0.001 compared with the negative control groups. A two‐tailed *t*‐test or ANOVA was used to assess the *P*‐values. Scale bars, 100 μm

To analyze the oncogenic character of YY1 in BCa, we knocked down *YY1* expression in both BCa cell lines (Figure 3A, Figure S2A). The results showed that deficiency of YY1 decreased the number of colony formation compared with that in the siNC group in MDA‐MB‐231 cells (Figure 3B), whereas cells transfected with the *YY1* overexpression vector produced more colonies in MDA‐MB‐231 cells (Figure S3C). Additionally, knockdown of *YY1* attenuated cell proliferation, as assessed using a CCK‐8 assay in MDA‐MB‐231 cells (Figure 3C), while overexpression of *YY*1 had the opposite effect (Figure S3B). Furthermore, knockdown of *YY1* suppressed migration and invasion of MDA‐MB‐231 cells (Figure 3D), whereas overexpression of *YY1* promoted cell migration and invasion (Figure S3D). Similar results were obtained using BT549 cells (Figure S2). Besides, to assess whether YY1 modulated the cell invasive growth by a KTN1dependent manner, MDA‐MB‐231 cells were treated with siKTN1 in the YY1 overexpression cells, which resulted in a fractional reduction of cell migration and invasion contrasted with YY1 overexpression alone group (Figure 3E, 3F). These results suggested that inhibition of YY1 could repress the proliferation and invasion of BCa.

To verify the pro‐carcinogenic YY1/*KTN1* axis in vivo, tumor‐bearing mice were injected with 5′ cholesterol‐ and 2′ methoxyethyl‐modified *YY1* siRNA oligos (10 nmol each) twice every week, and tumor volumes were recorded from 24 to 55 days after the injections. The results suggested that the volume of xenograft tumors treated with siYY1 oligos were significantly smaller than those injected with siNC oligos (*n* = 6 each, Figure 3G, 3H).

The mice were executed humanely, and their tumors were harvested for further assessment. Total RNA and protein were extracted to analyze their expression. The expression level of YY1 was effectively inhibited using siYY1 oligos treatment compared with that in the siNC group (Figure 3I). Next, Western blotting and IHC staining analysis showed that silencing of *YY1* markedly decreased the expression of KTN1 and mesenchymal markers, whereas it increased the expression of epithelial markers (Figure 3J, 3K). These data demonstrated that YY1 plays a pro‐carcinogenic role in invasive BCa. Targeting *YY1* with siRNA oligos might serve for a novel therapeutic approach to alleviate BCa progression.

### 2.4 The promoter of *KTN1* gene is directly transactivated by YY1

**FIGURE 4:**
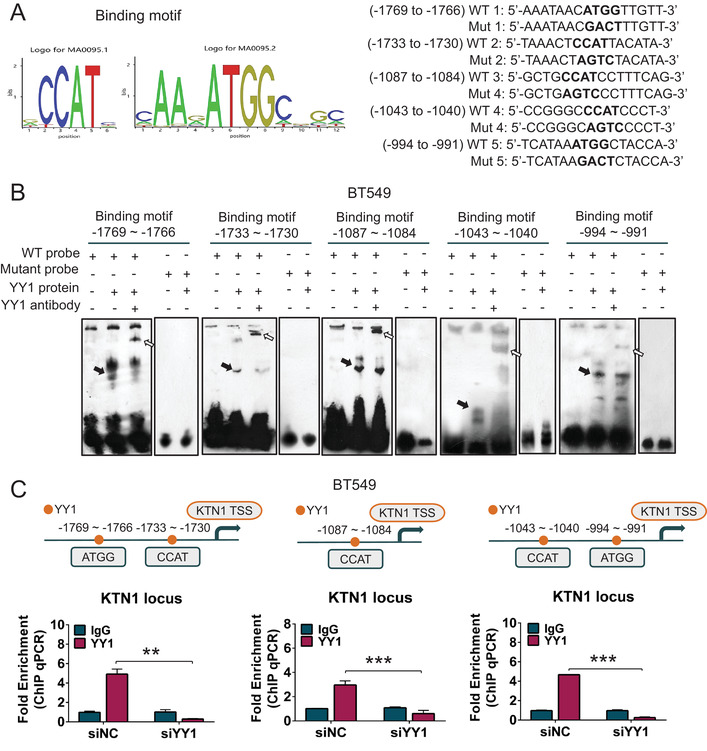
YY1 directly transactivated *KTN1* via binding to its promoter n BCa. (A) Using the UCSC and JASPAR databases analysis of predicted binding sites, including wild‐type (WT) and mutant (Mut) versions of these binding motifs. (B) Electrophoretic mobility shift (EMSA) assay analysis of the direct binding between the purified YY1 protein and the *KTN1* promoter. The black arrow represents the binding complex between YY1 and a probe, and the white arrow represents the supershift generated by the association of the anti‐YY1 antibody with YY1 and the probe. (C) ChIP assay analysis of YY1 enrichment on the promoter of *KTN1* in MDA‐MB‐231 cells compared with that using the immunoglobulin G mouse antibody. The different regions containing different putative YY1‐binding sites from left to right are shown. The binding enrichment of YY1 at the above binding sites on the promoter region was detected after knockdown of *YY1*. Error bars are shown with the s.d., *n* ≥ 3. * *P *< 0.05, ** *P *< 0.01, and *** *P *< 0.001. A two‐tailed *t*‐test or ANOVA was used to assess the *P*‐values

Given that high YY1 expression promoted the expression of *KTN1* in BCa cells, we identified putative YY1 binding sites through the JASPAR database (http://jaspardev.genereg.net), and also verified that the putative binding sites for YY1 could be matched with promoter regions of the *KTN1* gene for “CCAT” or “ATGG” sites by the UCSC genome browser database (http://genome.ucsc.edu/, Figure 4A). Next, to explore whether YY1 transcriptionally modulated *KTN1* gene expression, electrophoretic mobility shift (EMSA) assays were used to performe the direct interactions between the purified YY1 protein and the predicted *KTN1* promoter motifs, which was revealed by a super‐shift upon binding with the anti‐YY1 antibody. However, mutation of the YY1‐binding motifs within the *KTN1* promoter abolished the interaction, indicating the specificity of the binding sites for YY1 (Figure 4B, Figure S4A).

Additionally, a ChIP‐qPCR assay verified that the YY1 protein was recruited to the *KTN1* promoter at binding sites located at –1769 to –1766, –1733 to –1730, –1087 to –1084, –1043 to –1040, and –994 to –991, whereas signals was not detected at –1398 to –1395, –1379 to –1376, and –608 to –605 (Figure 4C, Figure S4B). Importantly, silencing of *YY1* decreased the recruitment of YY1 to the *KTN1* promoter markedly. Hence, these results indicated that YY1 specifically induced the transcriptional activity of the *KTN1* promoter at the above loci.

### 2.5 YY1‐mediated transactivation of *KTN1* gene promoter is co‐activated by DDX3X

**FIGURE 5:**
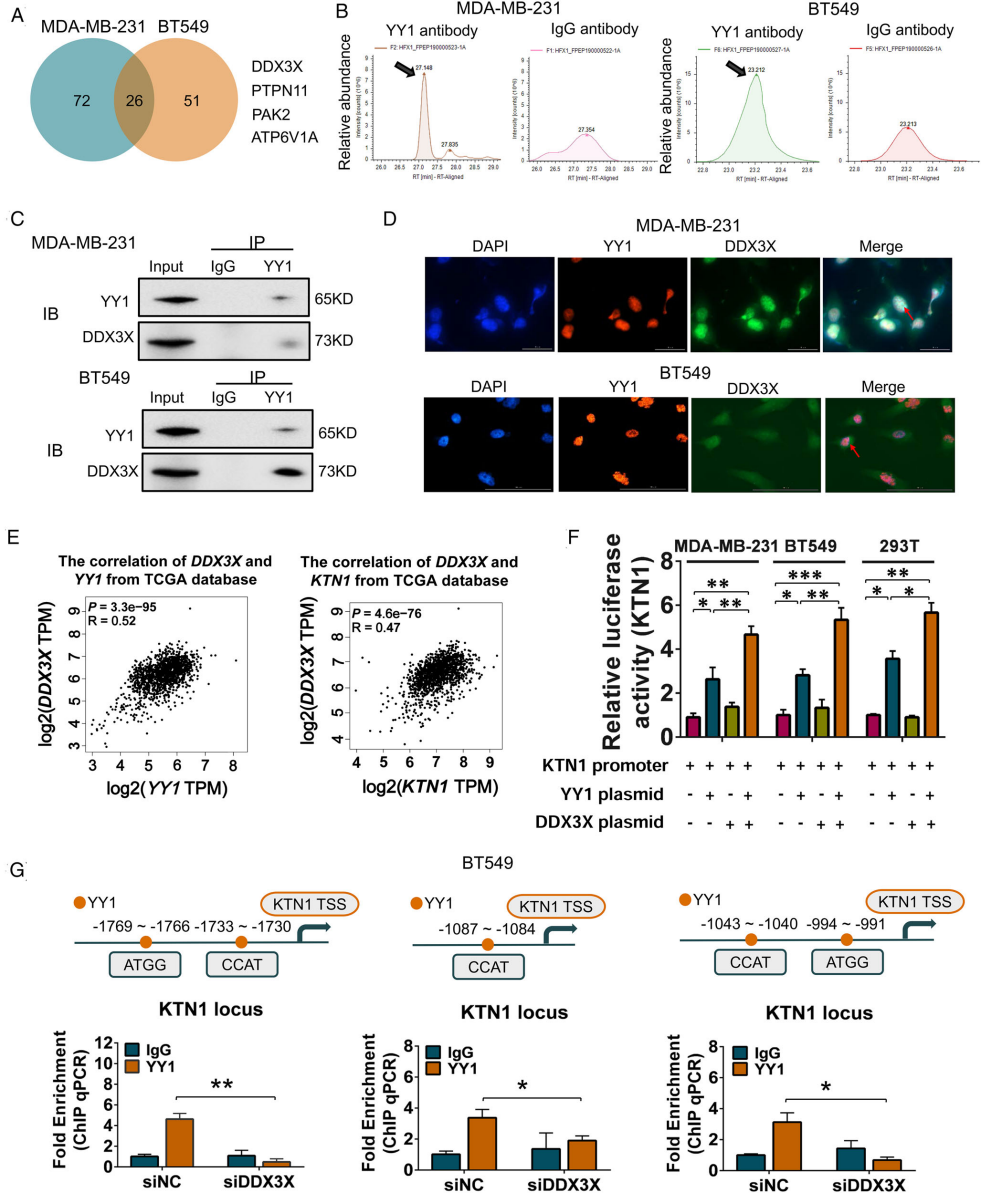
YY1 regulated the downstream target gene *KTN1* in a DDX3X‐dependent manner in BCa. (A) Chromatographic analysis of the proteins immunoprecipitated using anti‐YY1 antibodies or control IgG antibodies. (B) Black arrows indicate the DDX3X peptide peaks in the YY1‐pulldown samples from MDA‐MB‐231 and BT549 cells compared with those from control IgG samples. (C) Western blotting and co‐immunoprecipitation assays analysis of co‐factors of YY1**. (**D) Immunofluorescence chemistry assay analysis of the co‐location and co‐expression in both BCa cell lines. (E) The correlation analysis between DDX3X and YY1 expression from the GEPIA database (*R* = 0.52, *P *= 3.3e−95), and the correlation analysis between DDX3X and KTN1 expression from the GEPIA database (R = 0.47, *P *= 4.6e−76). (F) Activity of a reporter containing four canonical YY1‐binding sites and binding of YY1 to the *KTN1* promoter in MDA‐MB‐231, BT549, and 293T cells transfected with th*e KTN1* promoter reporter, the full‐length *DDX3X* plasmid, and the full‐length *YY1* plasmid (*n* = 4) as determined by a dual‐luciferase assays. (G) ChIP‐qPCR analysis showing the binding enrichment of YY1 at the binding sites on the promoter region of *KTN1* detected after knockdown of *DDX3X*. Error bars are showed with the s.d., *n* ≥ 3. *P < 0.05, **P < 0.01, and ***P < 0.001. A two‐tailed *t*‐test or ANOVA was used to assess the *P*‐values

YY1 is defined as a dual functional transcription factor, regulating downstream target genes via transcriptional activation or inhibition. This dual role of YY1 depends on its interacting partners.^13^ Considering the dual character of YY1, and to investigate the mechanistic role of its oncogenicity in BCa, co‐IP assays in combination with high‐performance liquid chromatography–mass spectrometry (HPLC–MS) analysis was performed to identify the proteins of interacting with YY1. In total, 72 (MDA‐MB‐231 cells, *H*/*L* ratio > 2.0) and 51 (BT549 cells, *H*/*L* ratio > 2.0) differentially abundant proteins were distinguished in the pull‐down assay using the YY1 protein as compared with the negative IgG group (Figure 5A). According to these candidate proteins, 28 (*H*/*L* ratio > 2.0) proteins were shared between MDA‐MB‐231 and BT549 cells. Potential YY1 cross‐linking proteins, including DDX3X, PTPN11, PAK2, and ATP6V1A proteins, were filtered through protein localization and outcome analysis using the human protein atlas online (https://www.proteinatlas.org/) and GEPIA databases (Figure 5B, Figure S5A, B). Western blotting analysis showed that DDX3X was verified as pulled down by YY1 (Figure 5C, Figure S5C) in both BCa cell lines. Therefore, the results suggested that DDX3X was a potential binding partner of YY1, corroborating its hypothetical role as a YY1 interaction co‐partner.

Knockdown of *DDX3X* inhibited lung metastasis in BCa,^19^ and DDX3X promoted cancer cells survival by modulating mRNA metabolism, the stress response, hypoxia, apoptosis, and the cell cycle.^25^ Immunofluorescence analysis showed that YY1 was co‐localized and co‐expressed with DDX3X in the nucleus of both BCa cell lines (Figure 5D). Consistently, we performed the correlation analysis of DDX3X, and YY1 or KTN1 in the TCGA database. The results suggested that the mRNA expression of *DDX3X* was related positively with the mRNA expression of *YY1* (*R* = 0.52, *P *= 3.3e−95, Figure 5E) and *KTN1* (*R* = 0.47, *P *= 4.6e−76, Figure 5E).

Next, to identify that whether DDX3X acted as a co‐activator of YY1 to regulate *KTN1* transactivation, we conducted the luciferase reporter assays including the binding motif of *KTN1* promoter. The data showed that the *KTN1* signal was excessively activated after co‐transfection of YY1 and DDX3X overexpression plasmids in three cell lines compared with other groups: the negative control group, the YY1 overexpression only group, and the DDX3X overexpression only group (Figure 5F). Using an anti‐YY1 antibody, we showed that depletion of DDX3X contributed to decreasing at the binding motifs of the *KTN1* promoter in YY1‐dependent manner (Figure 5G). These data suggested that DDX3X was required for YY1 transactivation the promoter of *KTN1*.

### 2.6 DDX3X augments YY1‐*KTN1* signaling axis‐promoted cell growth in high‐grade breast cancer

To demonstrate the oncogenic characteristics of DDX3X in BCa, the IHC results uncovered that the expression of DDX3X was increased in BCa tissues compared to patacancerous tissues (Figure S6A). Besides, high DDX3X expression was associated with poor RFS (*P *= 4.8e−06) in patients with invasive BCa, as indicated by Kaplan–Meier analysis (Figure S6B). QRT–PCR analysis also showed that the expression of *DDX3X* was increased in cancer cells compared to that in human mammary epithelial cells (Figure S6C). Furthermore, knockdown of *DDX3X* led to significantly downregulation of the levels of DDX3X and KTN1 proteins in MDA‐MB‐231 cell line (Figure S6D). To confirm if DDX3X blockade impacted the expression of KTN1, BT549 cells were treated with different concentrations of RK‐33 that was DDX3X inhibitor, and the expression of YY1, DDX3X, or KTN1 was assessed. We found that inhibition of DDX3X decreased the expression of KTN1 in no significant dose‐dependent manner, the reason was probably that decreasing of DDX3X activity weakened the capacity of YY1 at binding of KTN1 promoter. Moreover, the addition of YY1 overexpression was sufficient to partially rescue RK‐33 repressed expression of KTN1 (Figure S7). In addition, inhibiting the expression of *DDX3X* repressed the growth and invasion of MDA‐MB‐231 cells (Figure S6E, 6F).

Next, to evaluate whether YY1 regulated the cell invasive growth of BCa in a DDX3X‐dependent manner, BT549 cells with treated with siYY1 were transfected with the *DDX3X* overexpression plasmid, which resulted in a fractional rescue of cell migration and invasion contrasted to the siYY1 alone group (Figure S6G, S6H). Analogously, in *DDX3X* knockdown cells, we observed increased migration and invasion of cells following *YY1* overexpression contrasted to those in the siDDX3X alone group (Figure S6I, S6J). These results suggested that DDX3X was involved in YY1‐mediated BCa aggressive growth.

### 2.7 YY1 facilitates the aggressive growth of BCa tumors in vivo

**FIGURE 6:**
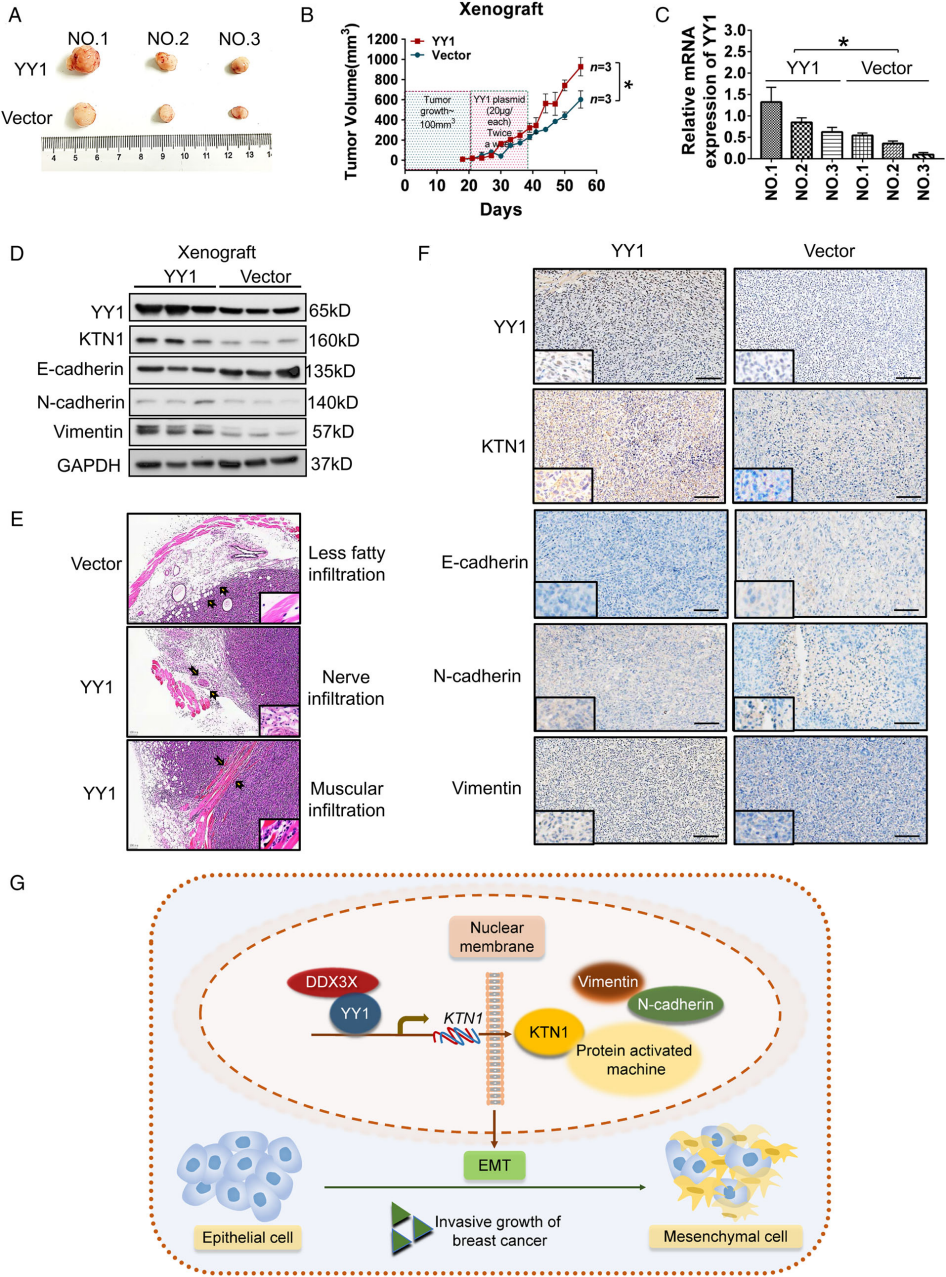
Overexpressed YY1 induced tumor aggressive growth in breast cancer (BCa). (A) Overexpression of *YY1* promoted MDA‐MB‐231 cell growth in mice xenograft models compared with that in the vector only group. (B) Tumor volumes were measured after injected MDA‐MB‐231 cells treated with the *YY1* overexpression plasmid in the xenograft mouse model (*n* = 3). (C) qRT‐PCR analysis of the expression levels of *YY1* mRNA in the xenograft tumors. (D) Western blotting analysis of the levels of YY1, KTN1, and epithelial‐to‐mesenchymal transition (EMT) markers. (E) Hematoxylin and eosin (HE) staining analysis of the effects on tumor cell aggression of *YY1* overexpression in vivo; a black arrow represents the range of infiltrating of tumor cells. (F) Immunohistochemistry (IHC) staining of YY1, KTN1, and EMT marker proteins in xenograft tumors. (G) A model of the YY1/DDX3X/*KTN1*‐regulatory axis in BCa development. Error bars are shown with the s.d., *n* ≥ 3. *P < 0.05, **P < 0.01, and ***P < 0.001 compared with the negative control group. A two‐tailed *t*‐test or ANOVA was used to assess the *P*‐values. Scale bars, 100 μm

To demonstrate whether YY1 promoted the aggressive growth of BCa tumors in vivo, we first evaluated the influence of *YY1* overexpression on BCa xenograft tumors using a NOD/SCID/IL2rγ null mice model (*n* = 3 each). When tumor bulk reached 100 mm^3^, the xenograft tumors were injected with *YY1* overexpression plasmids twice every week. The results suggested that the volume of xenografts treated with *YY1* overexpression plasmids were increased significant compared with those injected with the negative control vector (Figure 6A, 6B). In addition, overexpression of *YY1* in xenograft mice enhanced the expression of KTN1 and the mesenchymal markers (Vimentin and N‐cadherin), whereas it inhibited the epithelial protein expression (E‐cadherin) by western blotting (Figure 6C, 6D). IHC analysis indicated that the levels of mesenchymal markers were increased, whereas those of the epithelial marker decreased (Figure 6F). Importantly, H&E staining assessed the aggressive effects of *YY1* overexpression in vivo. The results indicated that *YY1* overexpression markedly promoted more tumor cells infiltration in adjacent fatty, nerve, and muscular tissues in contrast to that of the negative control vector groups (Figure 6E). Therefore, these findings demonstrated that YY1 promoted KTN1‐mediated the cell invasive growth of BCa, and YY1 transactivated the *KTN1* gene in a DDX3X‐dependent manner (Figure 6G).

## 3 DISCUSSION

As the most common cancer in women, high‐grade BCa often relapses, which is mainly attributed to the enhanced metastasis and invasive growth of cancer cells.^26^ Therefore, a comprehensive understanding of the molecular mechanisms modulating the process of cell invasion and metastasis is fundamental to improving the clinical outcome of BCa. The histological scores of BCa are assigned to three grades: Grade I, Grade II, and Grade III. Histological Grade III BCa tends to demonstrate an aggressive molecular biological signature, manifested as a “basal cluster”‐like gene expression profile, including high‐expression levels of *EGFR* (encoding epidermal growth factor receptor), *CK5* (cytokeratin 5), CK14, CK17, and vimentin.^27^, ^28^ Furthermore, patients with positive estrogen receptor (ER) expression are defined as Grade I to II and have a better prognosis for survival, while the patients with triple‐negative BCa usually suffer from Grade III BCa.^29^ Our previous study suggested that KTN1 promoted epithelial‐to‐mesenchymal transition (EMT) progression in triple negative BCa, whereas inhibition of *KTN1* expression could repress tumor EMT in vitro and in vivo.^9^

During cell development, proliferation, and oncogenicity, EMT progression represses epithelial characteristics and enhances the expression of certain genes that are characteristic of mesenchymal cells. In addition, EMT contributes to cancer occurrence and highly aggressive cancers.^30^ KTN1 acts as a membrane receptor that binds to kinesin protein. The KTN1–kinesin complex is involved in microtubule movement and organelle transport. Previous studies revealed that kinesin family members [Kinesin family member C1 (KIFC1), Kinesin family member 5B (KIF5B), and Kinesin light chain 1 (KLC1)] play important roles in accelerating epithelial−mesenchymal plasticity in bladder cancer and breast tumors.^31^, ^32^ High expression of KLFC1 promoted the phosphorylation of glycogen synthase kinase 3 beta (GSK3β) and enhanced the expression of *SNAI1* (encoding snail family transcriptional repressor 1) via protein kinase B (AKT) activation, which induced bladder cancer cell growth and EMT. Besides, in BCa, KIF5B and KLC1 promote epithelial−mesenchymal plasticity and tumorigenesis through regulating *TGFB* (encoding transforming growth factor beta) expression.^31^, ^32^ In the present study, we found that KTN1 could interact with KIF5B and KLC1 by analyzing data in the String database (https://string‐db.org/, Figure S7A). In addition, gene ontology (GO) analysis of the proteins interacting with KTN1 identified the “Kinesin complex” function (Figure S8). In addition, a Co‐IP assay analysis demonstrated that KIFC1 and KLC1 proteins could be pulled down by KTN1 antibody compared with negative control IgG groups in both types BCa cells (Figure S9). Thus, our results demonstrated that KTN1 might promote EMT progression of BCa by binding kinesin family members, which should be further explored in the future.

Accumulating evidence indicates that YY1 is involved in cancer progression. Patten et al. considered that YY1 acts a key element of ER alpha (ERα) transcriptional activity involving in luminal BCa growth, and could contribute to resistance to endocrine therapy.^33^ However, whether YY1 and its regulatory axis could promote cell invasion in advanced BCa was unknown. A previous report showed that oncogenic mechanistic target of rapamycin 2 (mTOR2)‐mediated AKT signaling was activated by YY1.^34^ In our study, YY1 was revealed as a crucial transcription factor that activates *KTN1* expression. In addition, upregulated *YY1* correlated positively with pathological progression and poor clinical outcome, which strongly indicated a pro‐oncogenic role of this gene in BCa. Gene loss‐of‐function and gain‐of‐function experiments further verified that YY1 was able to enhance the invasive cell growth of BCa both in vitro and in vivo. These results clarified that YY1 promotes BCa progression and might be a potential therapeutic target to treat advanced BCa. However, the molecular mechanisms of YY1‐induced EMT progression are not clear. Shu et al. found that overexpression of *YY1* promoted vascular endothelial growth factor (VEGFA) and SNAI1 expression, and enhanced high glucose‐stimulated EMT progression and cell permeability.^35^ However, our findings suggested that increased YY1 expression expedited tumor cell EMT and aggressive growth in high‐grade BCa by transactivating the *KTN1* gene.

Interestingly, as a transcription factor, YY1 exhibits bidirectional transcription regulation in certain tumor contexts. It can activate or repress the transcription of its target gene depending on the interacting transcriptional co‐factors.^14^ Therefore, we sought to clarify the regulatory axis of YY1 in BCa progression by identifying its specific interacting protein in advanced BCa cells. Co‐IP combined with HPLC–MS analysis identified that transcription factor DDX3X could be an important cofactor of YY1. We verified that these two proteins interact in BCa cells. DDX3X is encoded on the X chromosome and is widely expressed in human tissues.^36^ A previous study showed that DDX3X was modulated by hypoxia‐inducible factor 1 alpha (HIF‐1α) directly and thus promotes tumorigenesis of BCa.^37^ Our rescue experiments demonstrated that overexpression of *DDX3X* in *YY1*‐silenced BCa cells increased their cell proliferation and invasion, suggesting that DDX3X might endow a pro‐oncogenic role on YY1 in advanced BCa. Additionally, our findings identified that YY1 positively modulated the expression of *KTN1* in a DDX3X‐dependent manner in BCa.

In conclusion, we identified that the YY1‐DDX3X‐*KTN1* signaling axis was markedly upregulated in BCa, which correlated positively with its pathological grading and poor prognosis. Gene loss‐of‐function and gain‐of‐function studies suggested that the YY1‐*KTN1* signaling pathway accelerated the aggressive growth of BCa cells both in vitro and in vivo, which could be augmented by DDX3X, the specific transcriptional coactivator of YY1. These findings highlighted a progression‐promoting function of the YY1‐DDX3X‐*KTN1* transcription regulatory axis in BCa and indicated potential therapeutic targets to overcome this disease.

## 4 MATERIALS AND METHODS

### 4.1 Clinical samples, immunohistochemistry, and survival curve according to BCa pathological grades

For clinical samples, three commercial tissue microarrays of BCa were purchased and obtained from Shanghai Outdo Biotech Co., Ltd (#HBreD140Su03, #HBreD077Su01 406, and #HBreD075Bc01). The expression of KTN1 in different pathological grades of BCa was reanalyzed based on tissue microarray immunostaining, as reported previously.^9^ Briefly, a total of 206 tissue samples of BCa, from low‐grade to high‐grade pathology, were diagnosed in the clinic (stage I = 37; stage II = 141; and stage III = 40) and 77 adjacent normal tissue samples were acquired. All tissue samples were paraffin‐embedded and cut into slices for IHC. All tissue samples were performed to dehydrate with ethanol after deparaffinizi with xylene. After washing in phosphate buffer, the antigen retrieval was performed by the sodium citrate buffer (pH 6.0). Endogenous peroxidase activity was quenched using hydrogen peroxide solution. And nonspecific crosslinking was covered with avidin biotin blocking solution (Abcam, avidin biotin blocking kit, ab64212). The tissue microarrays were incubated with primary antibodies at 4°C overnight. Next day, these samples were performed in the corresponding secondary antibodies labeled with horseradish peroxidase for 30 min–1 h after washing with phosphate buffer saline (PBS) at room temperature. The samples were stained by 3,3′‐diaminobenzidine (DAB) staning (Abcam, DAB substrate kit, ab64238). In the end, all microarrays were counterstained with hematoxylin after washing by ultrafiltration water.

For the mouse experiments, xenograft masses were excised and fixed by 4% paraformaldehyde. The paraffin‐embedded slices were staining for IHC or hematoxylin and eosin (H&E) assays. The IHC staining score was plotted as mean of 95% confidence interval (95% CI) ± s.d., which used to estimate the range of parameters (^*^ *P *< 0.05, ^**^ *P *< 0.01, ^***^ *P *< 0.001). In the comparison of normal, well differentiated, moderately differentiated, and poorly differentiated tissues, the combinative sensitivity (intensity score) and specificity (proportion score) of targeted protein IHC was 95%CI (0, 3), 95%CI (4, 6), 95%CI (7, 9), and 95%CI (10, 12).^38^, ^39^ The staining score was determined as follows: intensity score (0, none; 1, weak; 2, intermediate; 3, strong), a proportion score (0, 0%; 1, 1%–25%; 2, 26%–50%; 3, 51%–75%; and 4, 76%–100%). The intensity score multiplied by the proportion score was defined as the total positive staining score.^40^

The mRNA correlation analysis was obtained from GEPIA online. Spearman rank analysis was performed to calculate the correlation between KTN1 and YY1 protein levels in the tissue microarrays. The survival prognosis of BCa was plotted by the Kaplan–Meier survival plotter online (www.kmplot.com).

### 4.2 Western blotting, antibodies, and reagents

Total cell lysates were isolated and subjected to western blotting as described previously.^41^ For western blotting assays, the antibodies comprised: the primary antibodies used were: anti‐YY1 [(Cell Signalling Technology (CST), Danvers, MA, USA, #63227, 1:3000), anti‐KTN1 (CST, #13243, 1:2000), anti‐DDX3X (Abcam, Cambridge, MA, USA, ab196032, 1:2000), anti‐β‐tubulin (CST, #2128, 1:2000), anti‐β‐actin (CST, #3700, 1:2000), anti‐glyceraldehyde‐3‐phosphate dehydrogenase (GAPDH; CST, #5174, 1:2000), anti‐Vimentin (CST, #5741, 1:2000), anti‐E‐cadherin (CST, #3195, 1:2000), anti‐N‐cadherin (CST, #13116, 1:2000), anti‐KIFC1 [Abclonal Technology (Abclonal), #A3304, 1:1000], and anti‐Kinesin light chain 1 (KLC1; Abclonal, #A3304, 1:1000). The following secondary antibodies were used: anti‐mouse IgG‐horseradish peroxidase (HRP, CST, #7076, 1:2000) and anti‐rabbit IgG‐HRP (CST, #7074, 1:2000). For chromatin immunoprecipitation‐quantitative real‐time PCR (ChIP‐qPCR), the antibodies comprised: anti‐YY1 (CST, Danvers, MA, USA, #63227, 10 μg), normal mouse IgG (Merck, Kenilworth, NJ, USA; #12‐371, 10 μg)) and normal rabbit IgG (Merck, #12‐370, 10μg). For double immunofluorescence, the antibodies comprised: goat anti‐Rabbit IgG (H+L) secondary antibody, dylight 488 (ThermoFisher, 35552), goat anti‐Mouse IgG (H+L) secondary antibody, dylight 594 (ThermoFisher, 35510). For immunoprecipitation (IP) assays, the antibodies comprised: anti‐YY1 (CST, Danvers, MA, USA, #63227, 10 μg), normal mouse IgG (Merck, Kenilworth, NJ, USA; #12‐371, 10 μg) and normal rabbit IgG (Merck, #12‐370, 10 μg). For immunofluorescence, the antibodies comprised: anti‐YY1 (CST, Danvers, MA, USA, #63227, 1:200), anti‐DDX3X (Abcam, Cambridge, MA, USA, ab196032, 1:200). The DDX3X inhibitor used was: RK‐33 (Selleck.cn, #S8246).

### 4.3 Xenograft model in vivo

The xenograft experiments were performed as previously described.^42^ For RNA interfering treatment, female BALB/c‐nude mice at 4–6 weeks old were purchased and obtained from the Medical Laboratory Centre of Guangdong Province (Guangzhou, China). Untreated MDA‐MB‐231 cells were surgically injected into the mammary fat pad of mice (1 × 10^7^ cells in Matrigel). Three to four weeks after cancer cell injection, cholesterol‐conjugated negative control small interfering RNA (siNC) or YY1 siRNA oligonucleotide (siYY1) was delivered into the xenograft tumor, respectively (All siRNA oligonucleotide were purchased from RiboBio Co. Ltd., Guangzhou, China). siNC or siYY1 (10 nmol) in 50 μl saline was used to treat the xenograft tumor mass once every 3 days for 4 weeks. For the invasive experiment of BCa, MDA‐MB‐231 cells (2×10^6^ in Matrigel) were delivered into the mammary fat pad of NOD/SCID/IL2rγ null mice. When tumor mass reached 100 mm^3^, these mice were executed treatment with 20 μg YY1 overexpression vector or equal control empty vector once every 3 days for 4 weeks. All tumor volumes were measured using the following formula: volume (mm^3^) = (length × width^2^)/2. The animals were sacrificed humanely, and the xenograft tumors were isolated, fixed with 4% paraformaldehyde for IHC staining.

### 4.4 Immunoprecipitation assay and high‐performance liquid chromatography–mass spectrometry analysis

The IP assay and HPLC–MS analysis were performed as previously described.^43^ Briefly, the cell lysates were extracted using a Pierce™ Classic Magnetic IP/Co‐IP kit (Thermo Fisher Scientific, Waltham, MA, USA) and performed to IP using 20 μg of YY1 antibodies. The sodium dodecyl sulfate polyacrylamide gel electrophoresis assay was used to detect precipitated protein complex that interacting with YY1. After band excision in silver staining and extraction and digestion, the digest was used to perform HPLC–MS analysis by the EASY‐nLC™ 1200 UHPLC system (Thermo Fisher Scientific) on an Orbitrap Q Exactive HF‐X mass spectrometer (Thermo Fisher Scientific). Each fraction was analyzed by Proteome Discoverer 2.2 (Thermo Fisher Scientific). The raw data reported in this article had been deposited in the OMIX, China National Center for Bioinformation/Beijing Institute of Genomics, Chinese Academy of Sciences (https://ngdc.cncb.ac.cn/omix/preview/MUpyKOZZ: accession no. OMIX979).^44^ The precipitated protein complex was also separated and identified by western blotting assay.

### 4.5 Electrophoretic mobility shift assay

The EMSA assay was conducted using a LightShift™ Chemiluminescent RNA EMSA kit (Thermo Fisher) following the manufacturer's instructions. Briefly, EMSA was performed using 5′ biotin‐labeled dsDNA probes (Thermo Fisher). EMSA was carried out using annealed DNA probes, purified YY1 protein (Abcam, ab152809, 3 μg), and anti‐YY1 antibodies (CST, Danvers, MA, USA, #63227, 1 μg). The images were acquired by the imaging system (Bio‐Rad, Hercules, CA, USA). The probes sequences are listed in the Supporting information (Table S4).

### 4.6 Chromatin immunoprecipitation and qPCR assay (ChIP–qPCR)

ChIP assay was tested using an EZ ChIP™ kit (Merck Millipore, Billerica, MA, USA) following the manufacturer's instructions, using 20 μg antibody against YY1 or negative control IgG (Merck Millipore). Total DNA was purified by phenol chloroform extraction and ethanol precipitation, and then diluted in nuclease‐free water. qPCR was performed using 1 μl of the immunoprecipitated samples and the SYBR Select Master Mix (Thermo Fisher). The fold enrichments of DNA signals were calculated compared with the IgG signals. Primers sequences are listed in Table S2.

### 4.7 Dual‐luciferase reporter assay

Dual‐luciferase reporter assay was performed using the manufacturer's instructions (Promega, Madison, WI, USA). In brief, the *KTN1* promoter sequence was obtained from genomic DNA and subcloned into vector pPRO‐RB‐Report (Ribobio Biotech Co., Ltd.), generating a pPRO‐RB‐Report‐KTN1 construct. Cells were cultured in 96‐well plates and transfected with pPRO‐RB‐Report‐KTN1 vector and/or pCMV‐YY1 plasmid, and/or pCMV‐DDX3X plasmid. The cells were extracted using lysis buffer after 48 h. The firefly luciferase activity and the Renilla luciferase activity were detected by the BioTek citation 5 system (BioTek, Winooski, VT, USA).

### 4.8 Statistics

All statistical analyses were performed by SPSS 21.0 software (IBM Corp., Armonk, NY, USA). Correlations between the expression levels of YY1, KTN1, or DDX3X and clinical prognosis of BCa patients were analyzed using the Pearson correlation test. Survival curves were plotted and analyzed by the Kaplan–Meier plotter by GraphPad Prism Software 7.0 (GraphPad Inc, La Jolla, CA, USA). All data were showed as the mean ± standard deviation (s.d.). All data were performed for normal distribution and homogeneity of variance. Means were compared using independent‐samples two‐tailed *t*‐tests or one‐way analysis of variance (ANOVA). *P < 0.05, ** *P *< 0.01, ***P < 0.001. Further detailed methods are provided in the Supporting information.

## CONFLICT OF INTERESTS

The authors declare that there is no conflict of interest that could be perceived as prejudicing the impartiality of the research reported.

## ETHICS STATEMENT

For the use of clinical tissues, this study was approved by the ethics committee of the Shenzhen People's Hospital and the tissues were obtained from patients of BCa who provided written informed consent for the surgical operation (Statement number: LL‐KY‐2020356). All the mouse experiments were approved by the ethics committee of the Second Clinical Medicine College of Jinan University (approval number 20200316‐23). The mice were handled in accordance with animal welfare regulations. All mice were sacrificed by cervical dislocation to avoid unnecessary pain.

## AUTHOR CONTRIBUTIONS

Lin Gao and N. Xie contributed to the design of this work, analysed the data, and drafted the manuscript. Junying Qiu, Jingyi Huang, Malin Hong, and Pan Zhao performed the experiments. Jingyi Huang, Zhe Zhang, and Jinquan Xia took charge of analysing the bioinformatic data. Yong Dai, Yuwei Luo, and Wenbin Zhou provided breast cancer specimens. Jing Jiang, Hui Gong, Jing Xu, Li Fu, and Jigang Wang supported the acquisition of the clinical and pathological information. Yong Dai, Dixian Luo, and Chang Zou designed and supervised this work and revised the manuscript.

## Supporting information

Supporting Information

## Data Availability

The dataset generated during the current study is available from the corresponding author on reasonable request.

## References

[1] Jiang YZ , Ma D , Suo C , et al. Genomic and transcriptomic landscape of triple‐negative breast cancers: Subtypes and treatment strategies. Cancer Cell. 2019;35(3):428‐440 e5. 10.1016/j.ccell.2019.02.001 30853353

[2] Bardia A , Parton M , Kummel S , et al. Paclitaxel with inhibitor of apoptosis antagonist, LCL161, for localized triple‐negative breast cancer, prospectively stratified by gene signature in a biomarker‐driven neoadjuvant trial. J Clin Oncol. 2018:JCO2017748392. 30235087 10.1200/JCO.2017.74.8392

[3] Liu YR , Jiang YZ , Xu XE , Hu X , Yu KD , Shao ZM . Comprehensive transcriptome profiling reveals multigene signatures in triple‐negative breast cancer. Clin Cancer Res. 2016;22(7):1653‐1662. 26813360 10.1158/1078-0432.CCR-15-1555

[4] Waks AG , Winer EP . Breast cancer treatment. JAMA. 2019;321(3):316. 30667503 10.1001/jama.2018.20751

[5] Ong LL , Lin PC , Zhang X , Chia SM , Yu H . Kinectin‐dependent assembly of translation elongation factor‐1 complex on endoplasmic reticulum regulates protein synthesis. J Biol Chem. 2006;281(44):33621‐33634. 16950774 10.1074/jbc.M607555200

[6] Kumar J , Yu H , Sheetz MP . Kinectin, an essential anchor for kinesin‐driven vesicle motility. Science. 1995;267(5205):1834‐1837. 7892610 10.1126/science.7892610

[7] Hu X , Xiang L , He D , et al. The long noncoding RNA KTN1‐AS1 promotes bladder cancer tumorigenesis via KTN1 cis‐activation and the consequent initiation of Rho GTPase‐mediated signaling. Clin Sci. 2021;135(3):555‐574. 10.1042/CS20200908 33480975

[8] Zhang Y , Gao L , Ma S , et al. MALAT1–KTN1–EGFR regulatory axis promotes the development of cutaneous squamous cell carcinoma. Cell Death Differ. 2019;26(10):2061‐2073. 30683916 10.1038/s41418-019-0288-7 PMC6748142

[9] Gao L , Chen S , Hong M , et al. Kinectin 1 promotes the growth of triple‐negative breast cancer via directly co‐activating NF‐kappaB/p65 and enhancing its transcriptional activity. Signal Transduct Target Ther. 2021;6(1):250. 34219129 10.1038/s41392-021-00652-x PMC8255318

[10] Shi Y , Seto E , Chang LS , Shenk T . Transcriptional repression by YY1, a human GLI‐Kruppel‐related protein, and relief of repression by adenovirus E1A protein. Cell. 1991;67(2):377‐388. 1655281 10.1016/0092-8674(91)90189-6

[11] Khachigian LM . The Yin and Yang of YY1 in tumor growth and suppression. Int J Cancer. 2018;143(3):460‐465. 29322514 10.1002/ijc.31255

[12] Thomas MJ , Seto E . Unlocking the mechanisms of transcription factor YY1: Are chromatin modifying enzymes the key? Gene. 1999;236(2):197‐208. 10452940 10.1016/s0378-1119(99)00261-9

[13] Sarvagalla S , Kolapalli SP , Vallabhapurapu S . The two sides of YY1 in cancer: A friend and a foe. Front Oncol. 2019;9:1230. 31824839 10.3389/fonc.2019.01230 PMC6879672

[14] Wottrich S , Kaufhold S , Chrysos E , Zoras O , Baritaki S , Bonavida B . Inverse correlation between the metastasis suppressor RKIP and the metastasis inducer YY1: Contrasting roles in the regulation of chemo/immuno‐resistance in cancer. Drug Resist Updat. 2017;30:28‐38. 28363333 10.1016/j.drup.2017.01.001

[15] Begon DY , Delacroix L , Vernimmen D , Jackers P , Winkler R . Yin Yang 1 cooperates with activator protein 2 to stimulate ERBB2 gene expression in mammary cancer cells. J Biol Chem. 2005;280(26):24428‐24434. 15870067 10.1074/jbc.M503790200

[16] Harbeck N , Huang CS , Hurvitz S , et al. Afatinib plus vinorelbine versus trastuzumab plus vinorelbine in patients with HER2‐overexpressing metastatic breast cancer who had progressed on one previous trastuzumab treatment (LUX‐Breast 1): An open‐label, randomised, phase 3 trial. Lancet Oncol. 2016;17(3):357‐366. 26822398 10.1016/S1470-2045(15)00540-9

[17] Mo J , Liang H , Su C , Li P , Chen J , Zhang B . DDX3X: Structure, physiologic functions and cancer. Mol Cancer. 2021;20(1):38. 33627125 10.1186/s12943-021-01325-7 PMC7903766

[18] Tantravedi S , Vesuna F . Targeting DDX3 in medulloblastoma using the small molecule inhibitor RK‐33. Transl Oncol. 2019;12(1):96‐105. 30292066 10.1016/j.tranon.2018.09.002 PMC6171097

[19] Xie M , Vesuna F , Botlagunta M , et al. NZ51, a ring‐expanded nucleoside analog, inhibits motility and viability of breast cancer cells by targeting the RNA helicase DDX3. Oncotarget. 2015;6(30):29901‐29913. 26337079 10.18632/oncotarget.4898 PMC4745771

[20] Chen HH , Yu HI , Cho WC , Tarn WY . DDX3 modulates cell adhesion and motility and cancer cell metastasis via Rac1‐mediated signaling pathway. Oncogene. 2015;34(21):2790‐2800. 25043297 10.1038/onc.2014.190

[21] Netanely D , Stern N , Laufer I , Shamir R . PROMO: An interactive tool for analyzing clinically‐labeled multi‐ohmic cancer datasets. BMC Bioinform. 2019;20(1):732. 10.1186/s12859-019-3142-5 PMC6933892 31878868

[22] Tang Z , Li C , Kang B , Gao G , Li C , Zhang Z . GEPIA: A web server for cancer and normal gene expression profiling and interactive analyses. Nucleic Acids Res. 2017;45(W1):W98‐W102. 28407145 10.1093/nar/gkx247 PMC5570223

[23] Wu S , Wang H , Li Y , et al. Transcription factor YY1 promotes cell proliferation by directly activating the pentose phosphate pathway. Cancer Res. 2018;78(16):4549‐4562. 29921695 10.1158/0008-5472.CAN-17-4047

[24] Chacon RD , Costanzo MV . Triple‐negative breast cancer. Breast Cancer Res. 2010;12(Suppl 2):S3. 10.1186/bcr2574 PMC2972557 21050424

[25] Bol GM , Xie M , Raman V . DDX3, a potential target for cancer treatment. Mol Cancer. 2015;14:188. 26541825 10.1186/s12943-015-0461-7 PMC4636063

[26] Liang Y , Zhang H , Song X , Yang Q . Metastatic heterogeneity of breast cancer: Molecular mechanism and potential therapeutic targets. Semin Cancer Biol. 2020;60:14‐27. 31421262 10.1016/j.semcancer.2019.08.012

[27] Irvin WJ Jr , Carey LA . What is triple‐negative breast cancer?. Eur J Cancer. 2008;44(18):2799‐2805. 19008097 10.1016/j.ejca.2008.09.034

[28] Takahashi H , Oshi M , Asaoka M , Yan L , Endo I , Takabe K . Molecular biological features of nottingham histological grade 3 breast cancers. Ann Surg Oncol. 2020;27(11):4475‐4485. 32436191 10.1245/s10434-020-08608-1 PMC7808708

[29] Adani‐Ife A , Amegbor K , Doh K , Darre T . Breast cancer in togolese women: Immunohistochemistry subtypes. BMC Womens Health. 2020;20(1):261. 33228656 10.1186/s12905-020-01130-2 PMC7686772

[30] Derynck R , Weinberg RA . EMT and Cancer: More than meets the eye. Dev Cell. 2019;49(3):313‐316. 31063750 10.1016/j.devcel.2019.04.026 PMC7672963

[31] Xiao KH , Teng K , Ye YL , et al. Kinesin family member C1 accelerates bladder cancer cell proliferation and induces epithelial–mesenchymal transition via Akt/GSK3beta signaling. Cancer Sci. 2019;110(9):2822‐2833. 31278883 10.1111/cas.14126 PMC6726677

[32] Moamer A , Hachim IY , Binothman N , Wang N , Lebrun JJ , Ali S . A role for kinesin‐1 subunits KIF5B/KLC1 in regulating epithelial mesenchymal plasticity in breast tumorigenesis. EBioMedicine. 2019;45:92‐107. 31204277 10.1016/j.ebiom.2019.06.009 PMC6642081

[33] Patten DK , Corleone G , Gyorffy B , et al. Enhancer mapping uncovers phenotypic heterogeneity and evolution in patients with luminal breast cancer. Nat Med. 2018;24(9):1469‐1480. 30038216 10.1038/s41591-018-0091-x PMC6130800

[34] Lin J , He Y , Wang B , et al. Blocking of YY1 reduce neutrophil infiltration by inhibiting IL‐8 production via the PI3K‐Akt‐mTOR signaling pathway in rheumatoid arthritis. Clin Exp Immunol. 2019;195(2):226‐236. 30229869 10.1111/cei.13218 PMC6330653

[35] Fu SH , Lai MC , Zheng YY , et al. MiR‐195 inhibits the ubiquitination and degradation of YY1 by Smurf2, and induces EMT and cell permeability of retinal pigment epithelial cells. Cell Death Dis. 2021;12(7):708. 34267179 10.1038/s41419-021-03956-6 PMC8282777

[36] Cannizzaro E , Bannister AJ , Han N , Alendar A , Kouzarides T . DDX3X RNA helicase affects breast cancer cell cycle progression by regulating expression of KLF4. FEBS Lett. 2018;592(13):2308‐2322. 29782654 10.1002/1873-3468.13106 PMC6100109

[37] Bol GM , Raman V , van der Groep P , et al. Expression of the RNA helicase DDX3 and the hypoxia response in breast cancer. PLoS One. 2013;8(5):e63548. 23696831 10.1371/journal.pone.0063548 PMC3656050

[38] Yang Y , Zhou L , Lu L , et al. A novel miR‐193a‐5p‐YY1‐APC regulatory axis in human endometrioid endometrial adenocarcinoma. Oncogene. 2013;32(29):3432‐3442. 22907428 10.1038/onc.2012.360

[39] Pyo JS , Kim DH , Yang J . Diagnostic value of CD56 immunohistochemistry in thyroid lesions. Int J Biol Markers. 2018;33(2):161‐167. 29799356 10.1177/1724600817748538

[40] Li RH , Tian T , Ge QW , et al. A phosphatidic acid‐binding lncRNA SNHG9 facilitates LATS1 liquid–liquid phase separation to promote oncogenic YAP signaling. Cell Res. 2021.31 (10):1088–1105. 34267352 10.1038/s41422-021-00530-9 PMC8486796

[41] Verma N , Muller AK , Kothari C , et al. Targeting of PYK2 synergizes with EGFR antagonists in basal‐like TNBC and circumvents HER3‐associated resistance via the NEDD4‐NDRG1 axis. Cancer Res. 2017;77(1):86‐99. 27793840 10.1158/0008-5472.CAN-16-1797

[42] Hou J , Zhou Y , Zheng Y , et al. Hepatic RIG‐I predicts survival and interferon‐alpha therapeutic response in hepatocellular carcinoma. Cancer Cell. 2014;25(1):49‐63. 24360797 10.1016/j.ccr.2013.11.011

[43] Wilson ID . High‐performance liquid chromatography–mass spectrometry (HPLC–MS)‐based drug metabolite profiling. Methods Mol Biol. 2011;708:173‐190. 21207290 10.1007/978-1-61737-985-7_10

[44] Chen T , Chen X , Zhang S , et al. The Genome Sequence Archive Family: Toward explosive data growth and diverse data types. Genom Proteom Bioinform. 2021.19(4):578–583. 10.1016/j.gpb.2021.08.001 PMC9039563 34400360

