# Supplementary material for: Yin Yang 1 promotes aggressive cell growth in high‐grade breast cancer by directly transactivating kinectin 1
Source: MedComm (2020). 2022 Jul 5;3(3):e133. doi: 10.1002/mco2.133 (PMC9253731; doi:10.1002/mco2.133)
Supplement: Supplementary file 1 — Supporting Information [file MCO2-3-e133-s001.pdf]

Supplementary Materials for

Yin Yang 1 promotes aggressive cell growth in high-grade breast cancer by directly transactivating KTN1

Lin Gao[1#], Wenbin Zhou[2#], Ni Xie[6#], Junying Qiu[3], Jingyi Huang[1], Zhe Zhang[1], Malin Hong[1,4], Jinquan Xia[1], Jing Xu[1], Pan Zhao[1,4], Li Fu[5], Yuwei Luo[2], Jing Jiang[7], Hui Gong[7], Jigang Wang[1,4], Yong Dai[1], Dixian Luo[7*], Chang Zou[1, 4, 8*]

Chang Zou (zouc2020@mail.sustech.edu.cn); Dixian Luo (luodixian_2@163.com)

This PDF file includes:

Supplementary Methods

Figures. S1 to S8

Report of Human Cell Line Authentication

Sequencing data acquisition

The data of Chromatin immunoprecipitation sequencing for from Richard Myers data (GEO:GSM803535, UCSC-ENCODE-hg19: wgEncodeEH001573) analysis were obtained from UCSC genome browser database (http://genome.ucsc.edu/). Kaplan-Meier survival analysis for the prognosis of breast cancer patients were performed with high or low YY1, KTN1, or DDX3X mRNA expression through using Kaplan-Meier database (www.kmplot.com).

Cell transfection

Selected MDA-MB-231 and BT549 cell lines were transfected with siNC oligonucleotides, *YY1* small interfering RNA (siRNA), *KTN1* siRNA, *NR3C1* siRNA, *ESR1* siRNA or *DDX3X* siRNA oligonucleotides (RIBOBIO Biotech CO., Ltd., Guangzhou, China). The cells were seeded and grown to 70–90% confluence in cultured dishes of different standards. Diluted Lipofectamine 3000 (Thermo Fisher Scientific, Waltham, MA, USA) in Opti-MEM medium was mixed with siRNA oligonucleotides and add to the cells. The cells were incubated for 2–4 days at 37 °C with 5% CO_2. The cells were then collected for subsequent analysis.

RNA extraction and Quantitative real-time reverse transcription PCR (qRT-PCR) analysis

Total RNA was extracted from cells using RNeasy Protect Mini Kit (QIAGEN) according to the manufacturer's instructions. Reverse transcription was conducted using TransScript One-Step RT-PCR SuperMix (Transgen Biotech, AT411-02, Beijing, China). Briefly, the mixtures containing total RNA, anchored Oligo (dT), 2×ES reaction mix, enzyme mix, gDNA remover and nuclease-free water were incubated 30 min at 42°C. Next, surplus enzyme mix and gDNA remover were inactivated at 85°C for 5 seconds (sec). Obtained cDNAs were used to performed quantitative real-time PCR (qPCR) reactions. qPCRassay was run using SYBR Green PCR Master Mix (Thermo Fisher Scientific) in a in 96-well plate (Bio-Rad, Hercules, CA, USA). Briefly, the mixtures containing cDNA templates (3μM), primers (10μM), SYBR Green PCR Master Mix (5μM) and nuclease-free water were performed to use one-step PCR procedure (94°C, 30sec; 94°C, 5sec; 60°C 30sec;40 cycles). The primer sequences are listed in the Table S1.

Cell culture

All cell lines were purchased from CellCook Biotech Co., Ltd. (Guangzhou, China). MCF10A cells were grown in Dulbecco's modified Eagle's medium (DMEM)/F12 medium (Gibco, Grand Island, NY, USA) supplemented with 5% horse serum (Gibco), 10 μg/ml insulin (Gibco), 20 ng/ml epidermal growth factor (EGF), 0.5 μg/ml hydrocortisone. MCF7 cells were cultured in minimal essential medium (MEM; Gibco) supplemented with 10% fetal bovine serum (FBS) and 10 μg/ml insulin, 1× nonessential amino acids (CellCook Biotech Co., Ltd.). ZR75-1 and HCC38 cells were grown in Roswell Park Memorial Institute (RPMI) 1640 medium supplemented with 10% FBS. T47D and BT459 cells were maintained in RPMI1640 medium supplemented with 10% FBS and 10 μg/ml insulin. MDA-MB-231 cells were maintained in DMEM medium supplemented with 10% FBS. All media were supplemented with 5,000 U/ml penicillin/streptomycin (Invitrogen, Waltham, MA, USA). All cells were maintained at 37 °C with 5% CO_2. All cell lines used in this study were authenticated using short tandem repeat typing (the results are presented in the Supporting information) and tested to exclude mycoplasma contamination.

Cell Proliferation and Colony Formation Assay

For the cell proliferation assay, MDA-MB-231(8000 per well) or BT549 cells (6000 per well) cultivated on 96-well plates (Corning Inc., Corning, NY, USA) were transfected with siRNA oligos, and cell proliferation was determined after 0, 24, 48, and 72 h using a Cell Counting Kit 8 (CCK-8) (MCE, Monmouth Junction, NJ, USA), with the results being recorded at 450 nm according to the manufacturers' instructions. For the cell colony formation assay, MDA-MB-231(1000 per well) or BT549 cells (1000 per well) were cultivated on 6-well plates. Attached cells were transfected with siRNA oligos, and cells were collected after 7 day. Cells were washed twice with phosphate-buffered saline (PBS) and fixed in 4% paraformaldehyde for 1 h. Cell colonies were photographed and counted after staining with 0.1% crystal violet.

Cell Migration and Invasiveness

For the cell migration assay, 1 × 10^5 cells treated with *YY1* or *KTN1* siRNA oligonucleotides, or overexpressing *YY1* from pCMV-YY1 vectors, were seeded in Transwell chambers (Merck Millipore,

Billerica, MA, USA; 8 μm) in 24-well plates (Corning Inc.). Complete medium (with 10% FBS) was added to the bottom of the Transwell chambers, while the serum-free medium was added to the top of the Transwell chambers. After being cultured for 16 to 20 h, the migrated cells were fixed with 4% paraformaldehyde, photographed, and counted for statistical analysis. Each experiment was repeated at least three times. For the cell invasion assay, 1×10^5 cells were seeded in Matrigel-coated Transwell chambers (BD, 8 μm) in 24-well plates. Other procedures were the same as the cell migration assay.

Double immunofluorescence (IFC) assay

The IFC assay was performed as previously described (1). Briefly, cells were fixed for 10 minutes using 4% paraformaldehyde at room temperature. The cells growing on glass coverslips were permeabilized using NP-40 reagent. The cells were blocked using bovine serum albumin (BSA) buffer for 15–20 minutes. The rabbit anti-YY1 and mouse anti-DDX3X primary antibody dilutions were added to cover the specimens and incubated at 4 °C overnight. Next day, the fluorescent secondary antibody and 4′,6-diamidino-2-phenylindole (DAPI) were incubated with the specimens, respectively. The slides were covered with ProLong™ Diamond (Thermo Fisher) antifade mountant and viewed under a fluorescence microscope.

Table S1. Sequences of qRT-PCR primers used in this study.

Primers	
**GAPDH**	Forward: 5′- ACATCGCTCAGACACCATG-3′
	Reverse: 5′- TGTAGTTGAGGTCAATGAAGGG-3′
**ACTB (β-Actin)**	Forward: 5′- ACCTTCTACAATGAGCTGCG-3′
	Reverse: 5′- CCTGGATAGCAACGTACATGG-3′
**YY1**	Forward: 5′- CTGGCATTGACCTCTCAGATC-3′
	Reverse: 5′- GCCGAGTTATCCCTGAACATC-3′
**KTN1**	Forward: 5′- TGGAATCAGAGCAGAAAAGGG-3′
	Reverse: 5′- GAACTGAAGCGGAGGTCTG-3′
**DDX3X**	Forward: 5′- CTGATCGGATGTTGGATATGGG-3′
	Reverse: 5′- TCTAAGAAATCACGAGCCAGC-3′
**ESR1**	Forward: 5′- CGACTATATGTGTCCAGCCAC-3′
	Reverse: 5′- CCTCTTCGGTCTTTTCGTATCC-3′
**NR3C1**	Forward: 5′- CCCGTTGGTTCCGAAAATTG-3′
	Reverse: 5′- AGCTTACATCTGGTCTCATGC-3′

Table S2. Sequences of ChIP-qPCR primers used in this study.

Primers

**KTN1-YY1_1**	Forward: 5’- CAAAGAGCAAACGAATAAGGAGAG-3’<br>Reverse: 5’- GAAAGCGACCCAAATGAGTTG-3’
**KTN1-YY1_2**	Forward: 5’- AACCTGCGTTAGTACTAACCTG-3’<br>Reverse: 5’- CCAGAAGATCCAGCTTTGTTG-3’
**KTN1-YY1_3**	Forward: 5’- GGAGGTGCTCTAGAAAACTTA-3’<br>Reverse: 5’- CCTACATCTGACAAAGTGGGA-3’
**KTN1-YY1_4**	Forward: 5’- ACAGCTTGGAGTGTTTTGTACA-3’<br>Reverse: 5’- TCCTTATTCGTTTGCTCTTTGCT-3’
**KTN1-YY1_5**	Forward: 5’- ACCTCAGGTGATCCGCCT-3’<br>Reverse: 5’- TCTGAAATCTCCTTCGGGCA-3’

Table S3. Sequences of siRNAs used in this study.

	**Sequences**
**siYY1_1**	5’- CCTGAAATCTCACATCTTA-3’
**siYY1_2**	5’- GATGGTTGTAATAAGAAGT-3’
**siDDX3X_1**	5’- CATTGAGCTTACTCGTTAT-3’
**siDDX3X_2**	5’- CTACGATAAAGACAGTTCA-3’
**siKTN1**	5’- GAGTGATCTTTCTAGCAAA-3’
**siNR3C1**	5’- AGGGCCAAATCAGCCTTTC-3’
**siESR1**	5’- GGAAGTATGGCTATGGAAT-3’

Table S4. Sequences of EMSA probes used in this study.

	**Sequences (Wild-type)**
**c-YY1 motif_KTN1 locus_top_1**	5’- CTGAGGAAATAACATGGTTGTTTGTA-3’
**c-YY1 motif_KTN1 locus_bottom_1**	5’- TACAAACAACCATGTTATTTCCTCAG-3’
**c-YY1 motif_KTN1 locus_top_2**	5’- GCCCTTAAACTCCATTACATAAATGA-3’
**c-YY1 motif_KTN1 locus_bottom_2**	5’- TCATTTATGTAATGGAGTTTAAGGGC-3’
**c-YY1 motif_KTN1 locus_top_3**	5’- AATAAAAAGTATGGATACTTTTAACC-3’
**c-YY1 motif_KTN1 locus_bottom_3**	5’- GGTTAAAAGTATCCATACTTTTTATT-3’
**c-YY1 motif_KTN1 locus_top_4**	5’- TTAACCAGCCCATGTACAGGAAAT-3’
**c-YY1 motif_KTN1 locus_bottom_4**	5’- ATTTCCTGTACATGGGCTGGTTAA-3’
**c-YY1 motif_KTN1 locus_top_5**	5’- GCTGCCATCCTTTCAGCAAA-3’
**c-YY1 motif_KTN1 locus_bottom_5**	5’- TTTGCTGAAAGGATGGCAGC-3’
**c-YY1 motif_KTN1 locus_top_6**	5’- AGATCCGGGCCCATCCCT-3’
**c-YY1 motif_KTN1 locus_bottom_6**	5’- AGGGATGGGCCCGGATCT-3’
**c-YY1 motif_KTN1 locus_top_7**	5’- GCTTCATAAATGGCTACCAGCT-3’
**c-YY1 motif_KTN1 locus_bottom_7**	5’- AGCTGGTAGCCATTTATGAAGC-3’
**c-YY1 motif_KTN1 locus_top_8**	5’-CGCCCGACCATTATTTGGGT -3’
**c-YY1 motif_KTN1 locus_bottom_8**	5’- ACCCAAATAATGGTCGGGCG-3’
	**Sequences (Mutant type)**
**c-YY1 motif_KTN1 locus_top_1**	5’- CTGAGGAAATAACGACTTTGTTTGTA-3’
**c-YY1 motif_KTN1 locus_bottom_1**	5’- TACAAACAAAGTCGTTATTTCCTCAG -3’
**c-YY1 motif_KTN1 locus_top_2**	5’- GCCCTTAAACTAGTCTACATAAATGA -3’
**c-YY1 motif_KTN1 locus_bottom_2**	5’- TCATTTATGTAGACTAGTTTAAGGGC -3’
**c-YY1 motif_KTN1 locus_top_3**	5’- AATAAAAAGTGACTATACTTTTAACC -3’
**c-YY1 motif_KTN1 locus_bottom_3**	5’- GGTTAAAAGTATAGTCACTTTTTATT -3’

**c-YY1 motif_KTN1 locus_top_4**	5’- TTAACCAGCAGTCGTACAGGAAAT -3’
**c-YY1 motif_KTN1 locus_bottom_4**	5’- ATTTCCTGTACGACTGCTGGTTAA -3’
**c-YY1 motif_KTN1 locus_top_5**	5’- GCTGAGTCCCTTTCAGCAAA -3’
**c-YY1 motif_KTN1 locus_bottom_5**	5’- TTTGCTGAAAGGGACTCAGC -3’
**c-YY1 motif_KTN1 locus_top_6**	5’- AGATCCGGGCAGTCCCCT -3’
**c-YY1 motif_KTN1 locus_bottom_6**	5’- AGGGGACTGCCCGGATCT -3’
**c-YY1 motif_KTN1 locus_top_7**	5’- GCTTCATAAGACTCTACCAGCT -3’
**c-YY1 motif_KTN1 locus_bottom_7**	5’- AGCTGGTAGAGTCTTATGAAGC -3’
**c-YY1 motif_KTN1 locus_top_8**	5’- CGCCCGAAGTCTATTTGGGT -3’
**c-YY1 motif_KTN1 locus_bottom_8**	5’- ACCCAAATAGACTTCGGGCG -3’

References

[1] K. Im, S. Mareninov, M.F.P. Diaz, W.H. Yong. An Introduction to Performing Immunofluorescence Staining. Methods Mol Biol 2019;1897:299-311.

Supplementary Figure. S1

A

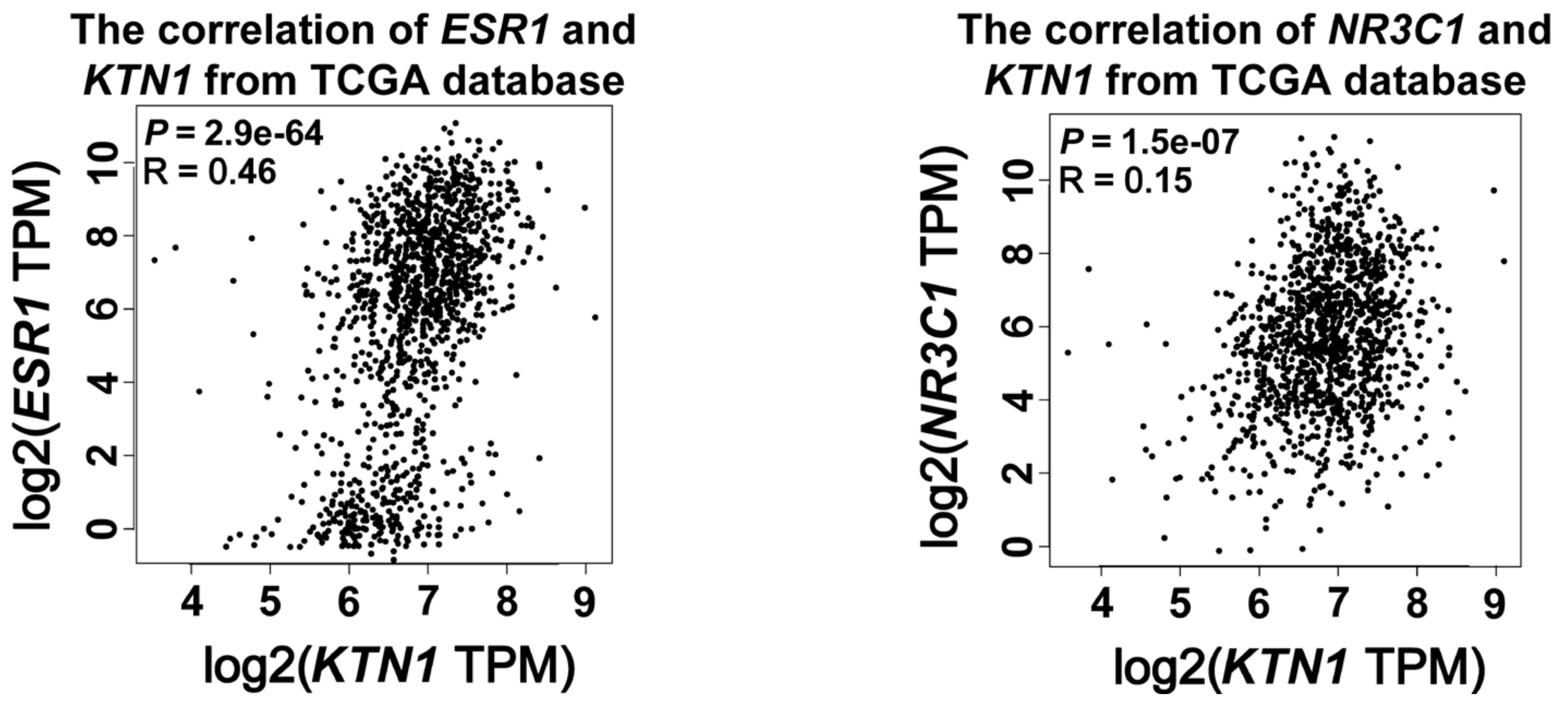

B

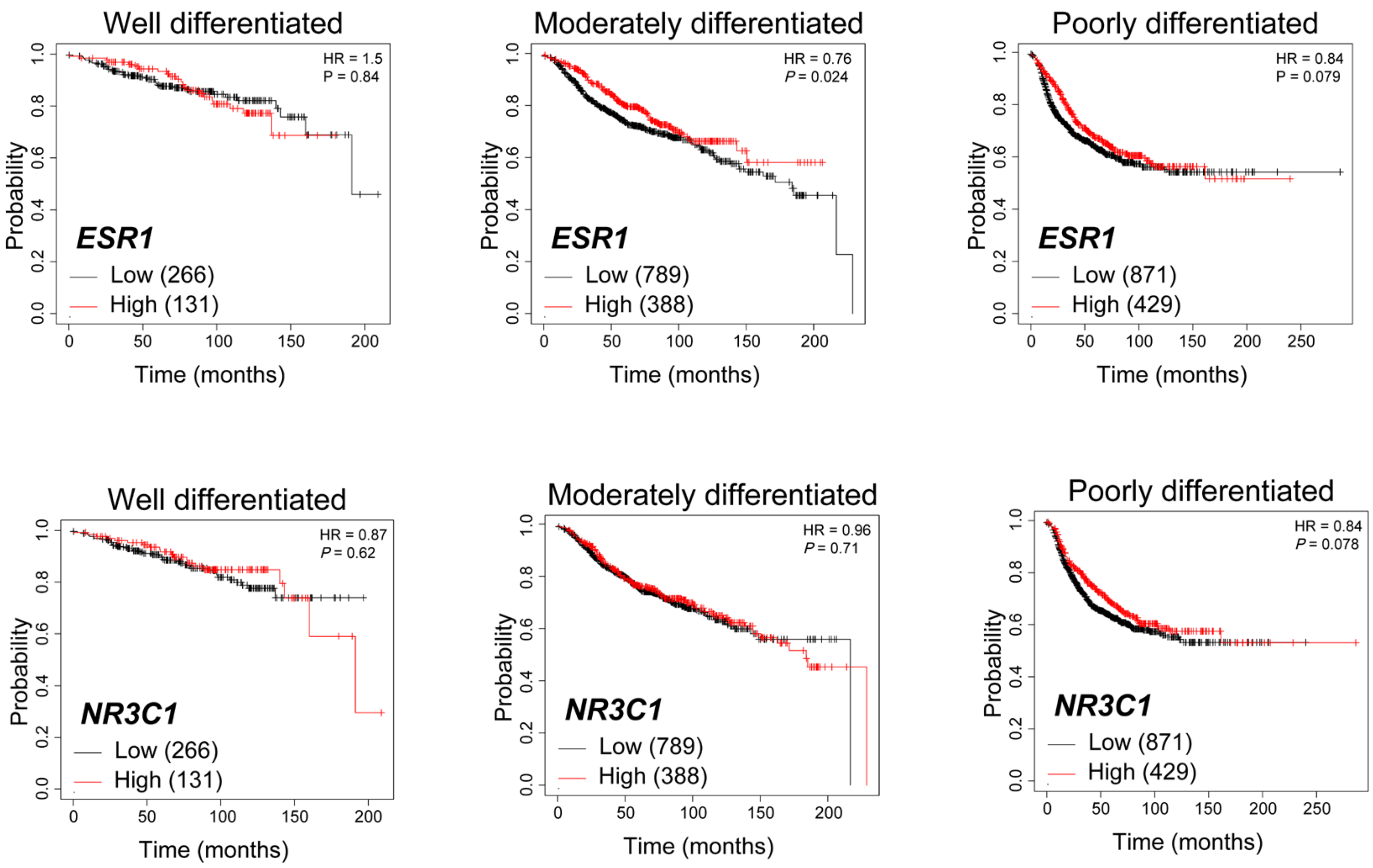

Supplementary Figure S1 Screening for transcription factors of *KTN1* gene. (A) The correlation analysis between ESR1 and KTN1, or NR3C1 and KTN1 mRNA from the TCGA database. **(B)** The relapse-free (RFS) analysis of BCa patients from the Kaplan-Meier plotter database (www.kmplot.com). Patients of BCa were ranked into 'high' (red line) and 'low' (black line) ESR1 or NR3C1 mRNA expression and showed the degree of different differentiation.

Supplementary Figure. S2

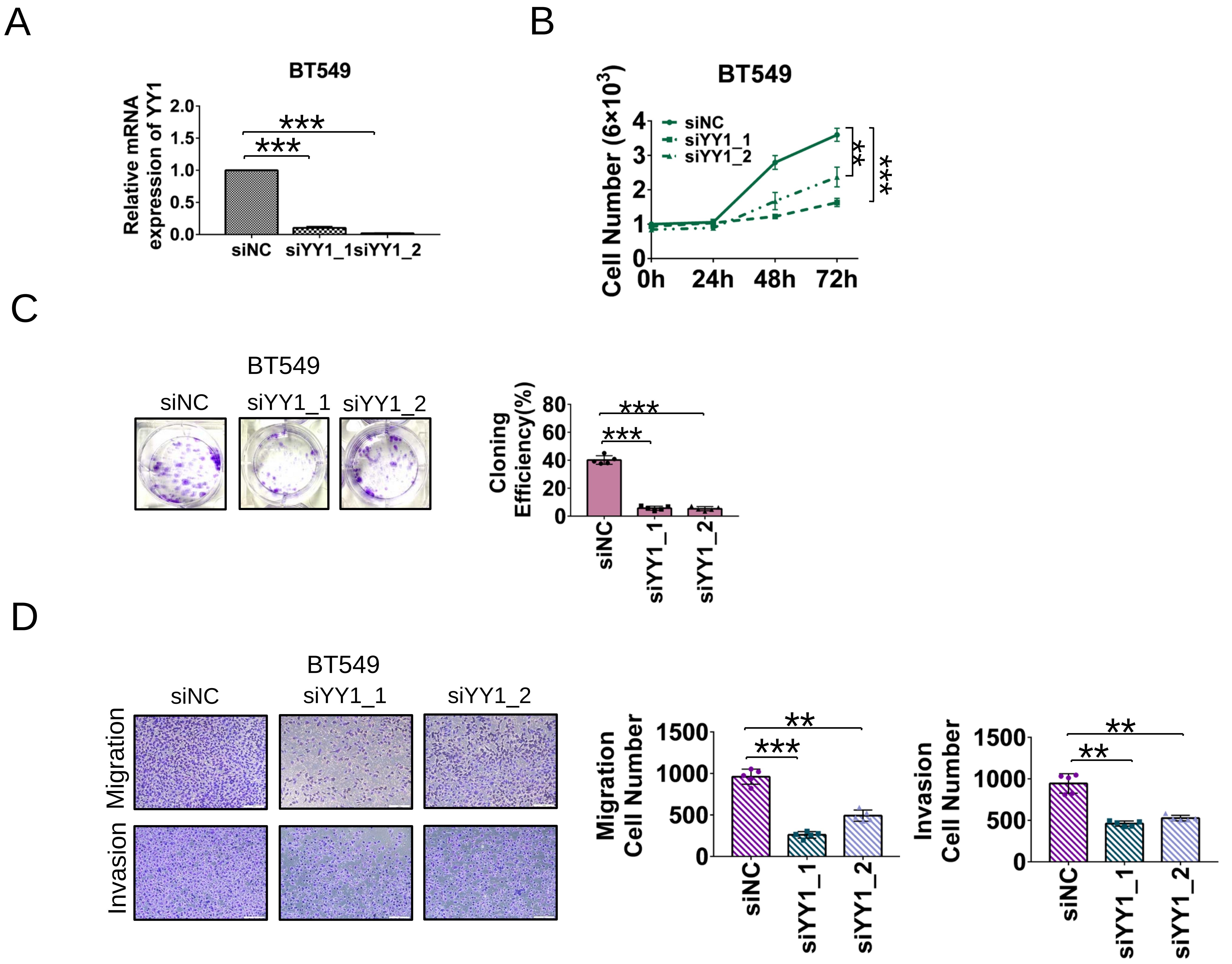

Supplementary Figure S2 The knockdown of YY1 inhibited the growth and invasion of BT549 cells. (A) YY1 knockdown in BT49 cells by YY1_1 and YY1_2 siRNA oligos was detected by qRT-PCR compared with negative control siRNA (siNC) oligos. **(B)** Measurement of cell proliferation by CCK-8 assay after cells treated with NC, siRNA oligos. **(C)** Measurement of cell colony forming by colony formation assay after cells treated with NC, siRNA oligos. **(D)** Transwell assay analysis of migration and invasion after cells treated with NC, siRNA oligos. Error bars was shown by s.d., n≥3. * *P* < 0.05, ** *P* < 0.01 and *** *P*< 0.001 were showed compared with the negative control groups. The two-tailed t-test or ANOVA was used to assess *P*-values. Scale bars, 100 μm.

Supplementary Figure. S3

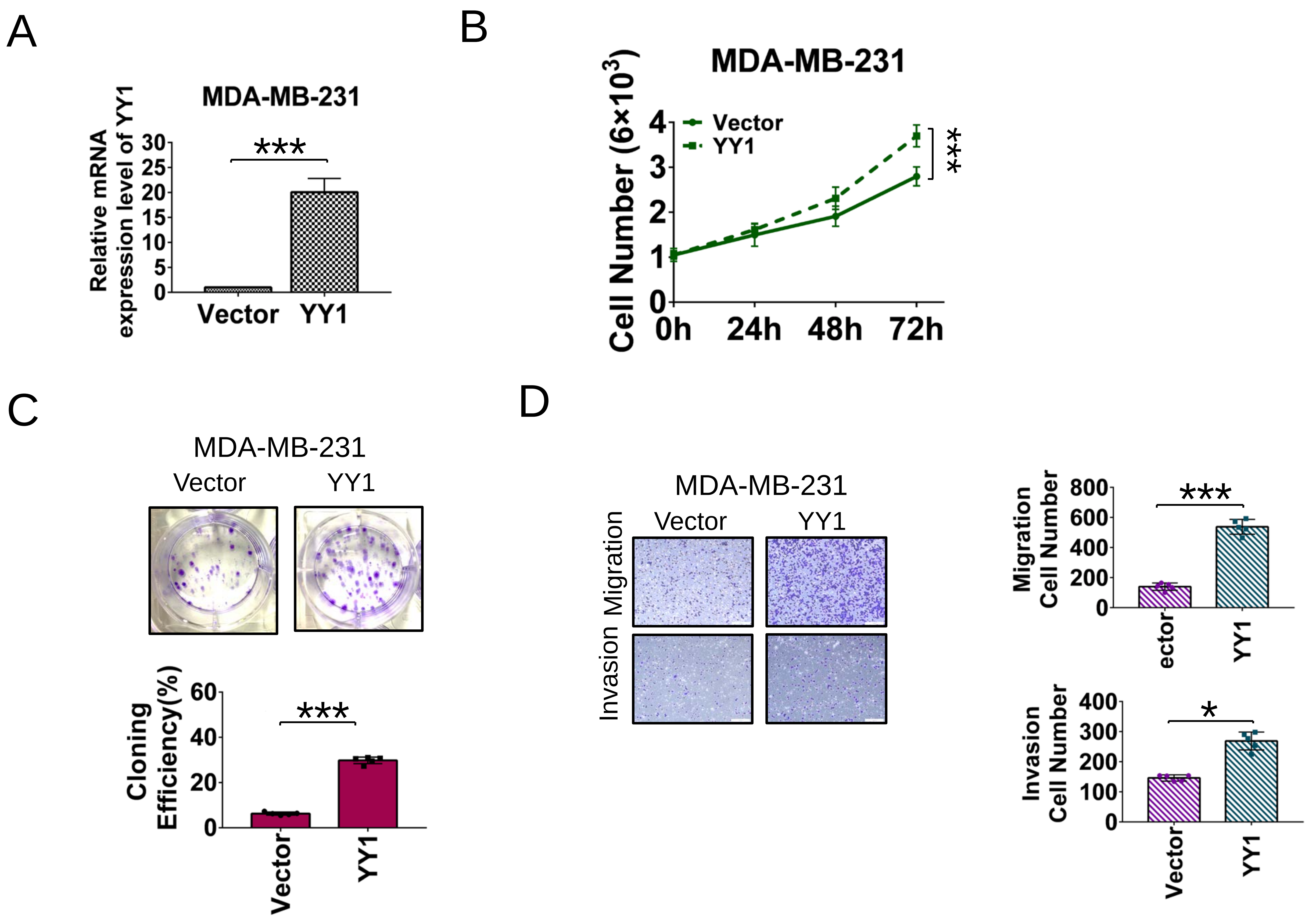

Supplementary Figure S3 YY1 promoted cell proliferation, migration, and invasion of BCa *in vitro*. (A) QRT-PCR analysis of the mRNA expression levels YY1 in MDA-MB-231 cells treated with overexpressed plasmid of Vector or YY1. **(B)** YY1 overexpression promoted cell proliferation by CCK-8 assay at 0h, 24h, 48h, and 72h. **(C)** YY1 overexpression conferred cell colony formation by colony formation assay. **(D)** YY1 overexpression facilitated tumor cell migration and invasion by Transwell assays. Error bars was shown by s.d., n≥3. * $P < 0.05$, ** $P < 0.01$ and *** $P < 0.001$ were showed compared with the negative control groups. The two-tailed t-test or ANOVA was used to assess *P*-values. Scale bars, 100 μm.

Supplementary Figure. S4

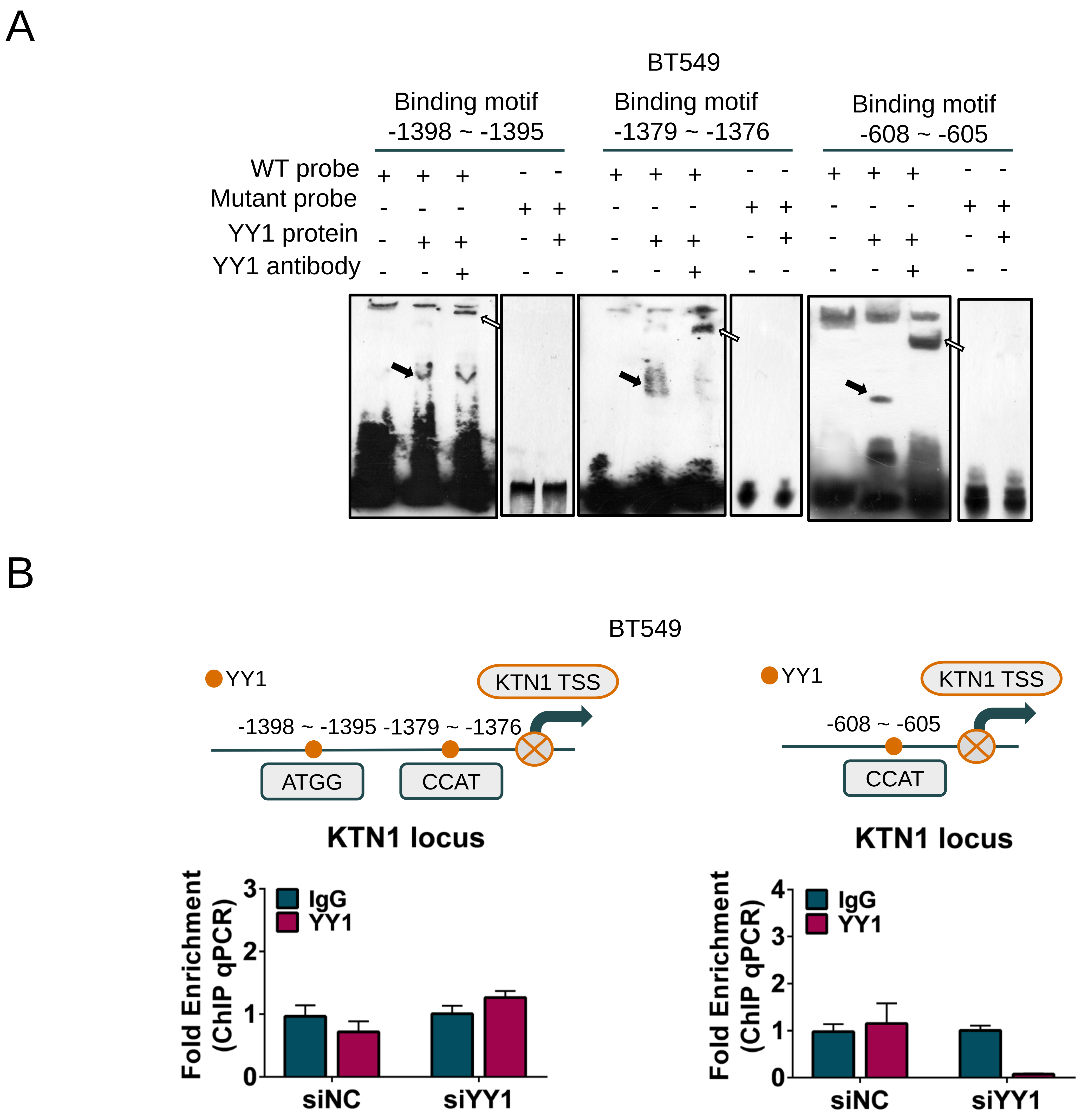

Supplementary Figure S4 YY1 bond to the promoter of *KTN1* gene. (A) EMSA assay analysis of the direct binding between purified YY1 protein and the *KTN1* promoter. The black arrow represented the binding complex between YY1 protein and a probe, and the white arrow represented the supershift generated by the association of anti-YY1 antibody with YY1 protein and probe. **(B)** ChIP assay analysis of YY1 enrichment on the promoter of *KTN1* gene in BT549 cells. IgG mouse, immunoglobulin G mouse. The different regions containing different putative YY1-binding sites from left to right shown. The binding enrichment of YY1 at the above binding sites on the promoter regions was detected after knockdown of YY1. Error bars was shown by s.d., n≥3. * $P < 0.05$, ** $P < 0.01$ and *** $P < 0.001$ were showed compared with the negative control groups. The two-tailed t-test or ANOVA was used to assess *P*-values.

Supplementary Figure. S5

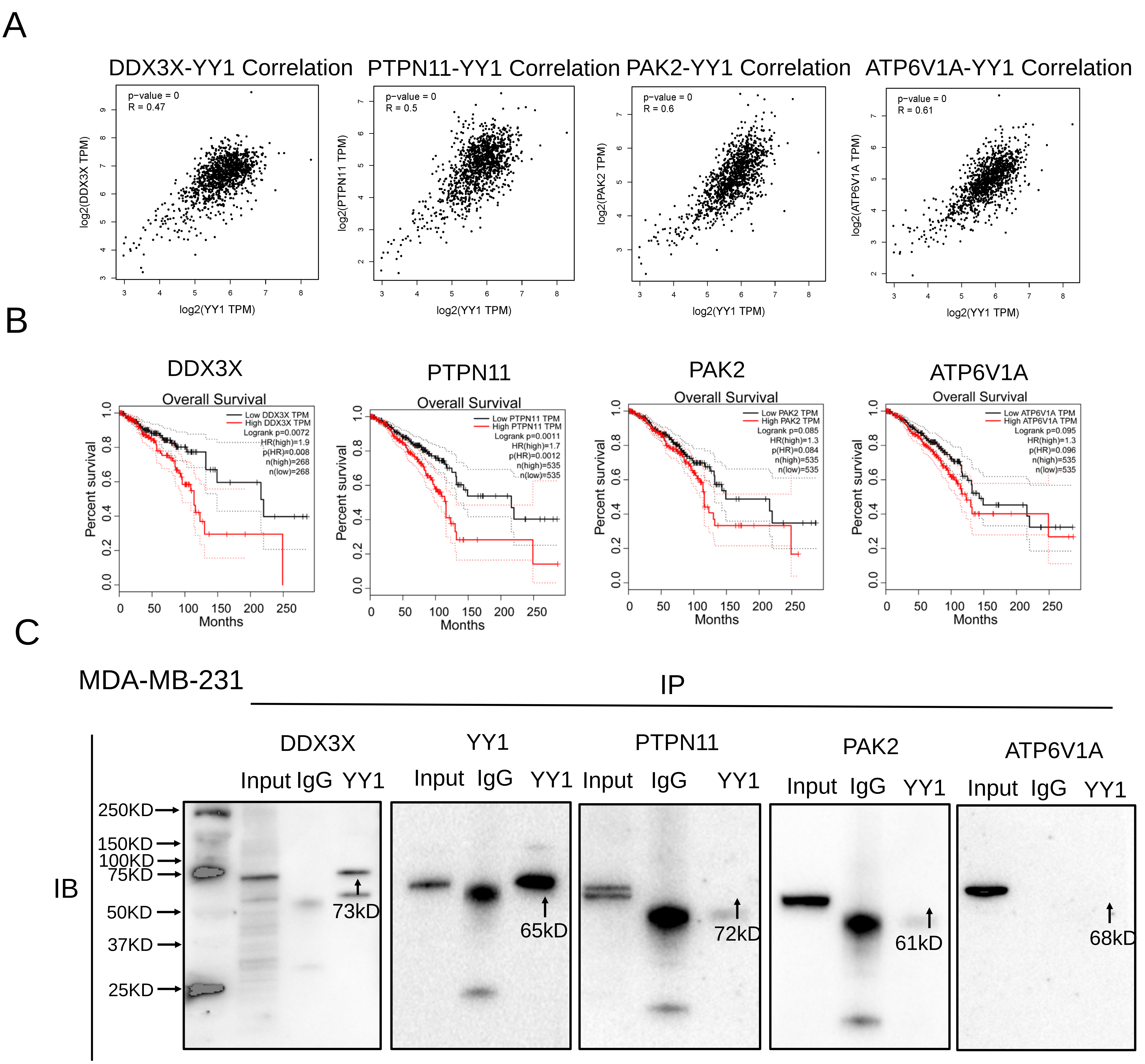

Supplementary Figure S5 Screening for co-activators that interact with YY1 protein. (A) The correlation analysis between YY1 and DDX3X, PTPN11, PAK2, ATP6V1A mRNA from the TCGA database. **(B)** Overall survival of BC patients from the GEPIA plotter database. *P*-values were calculated with log-rank (Mantel-Cox) test. Patients were grouped into 'low' (black) and 'high' (red) gene expression. **(C)** Co-immunoprecipitation (IP) followed with and western blot assay analysis of the proteins interacting with YY1 protein.

Supplementary Figure. S6

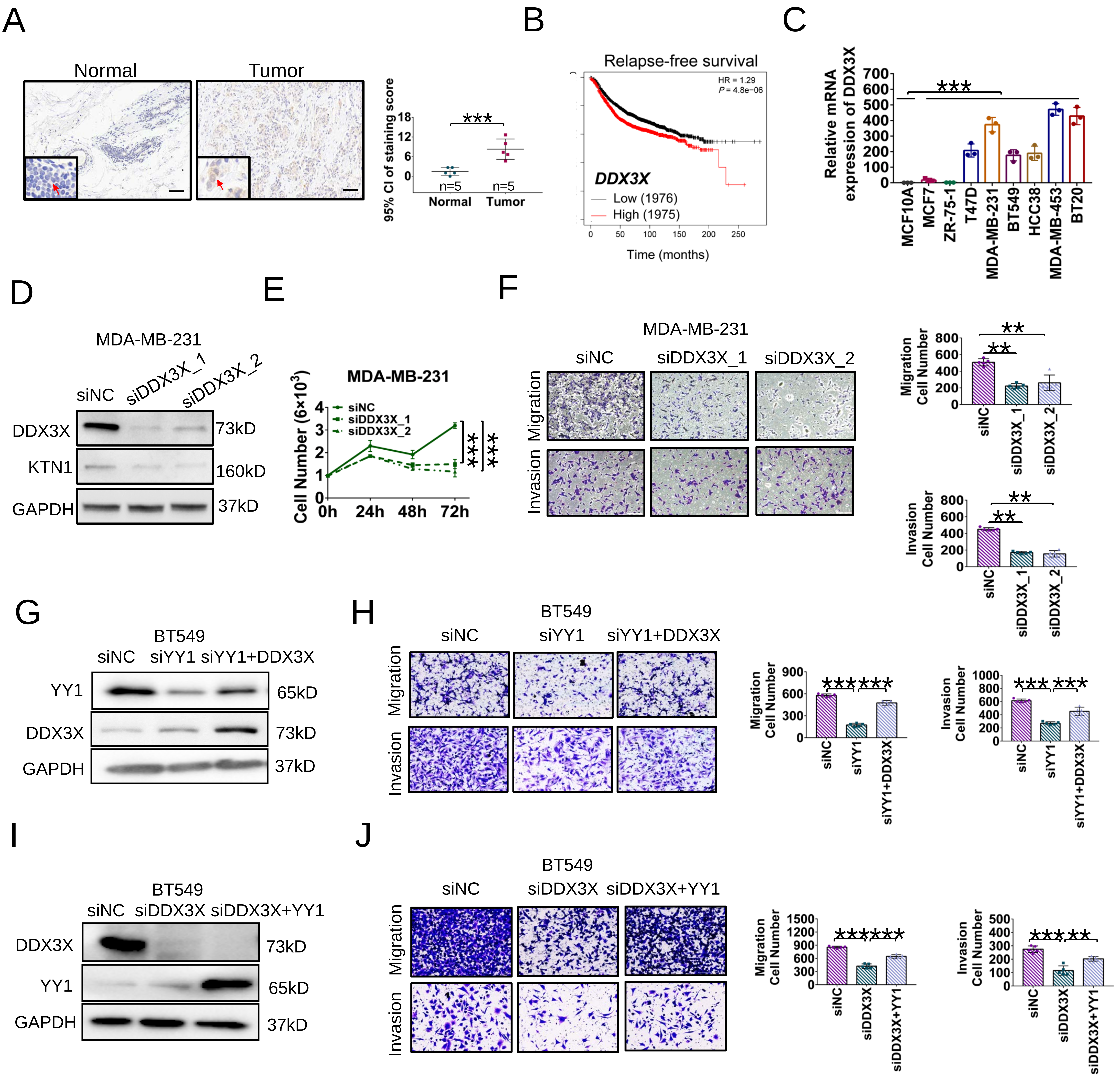

Supplementary Figure S6 The tumorigenesis of BCa by YY1 interacting with DDX3X protein. **(A)** IHC analysis of the expression of DDX3X. Red arrows indicated positive signals. **(B)** Kaplan-Meier analysis of RFS in BCa patients with high versus low expression levels of DDX3X mRNA from Kaplan-Meier plotter database. **(C)** QRT-PCR assays analysis of the expression levels of DDX3X mRNA in MCF10A, MCF-7, T47D, ZR75-1, MDA-MB-453, HCC38, BT20, MDA-MB-231, and BT549 cell lines. **(D)** Western blotting analysis of the expression levels of DDX3X and KTN1 in both BCa cell lines treated with siNC or siDDX3X oligos. **(E)** CCK-8 assay showing that proliferation of MDA-MB-231 cells treated with siNC or siDDX3X oligos. **(F)** Transwell assay showing that migration and invasion MDA-MB-231 cells treated with siNC or siDDX3X oligos. **(G)** Western blot analysis of YY1-deficienoft BT549 cells transfected with DDX3X-overexpressed plasmid. **(H)** Migration and invasion analysis of YY1-deficient BT549 cells transfected with DDX3X-overexpressed plasmid using Transwell assays. **(I)** Western blotting analysis of DDX3X-deficient BT549 cells transfected with the YY1 overexpression plasmid. **(J)** Migration and invasion analysis of DDX3X-deficient BT549 cells transfected with the YY1 overexpression plasmid using Transwell assays. Error bars are shown with the s.d., $n \geq 3$. * $P < 0.05$, ** $P < 0.01$ and *** $P < 0.001$. A two-tailed t-test or ANOVA was used to assess the P-values.

Supplementary Figure. S7

A

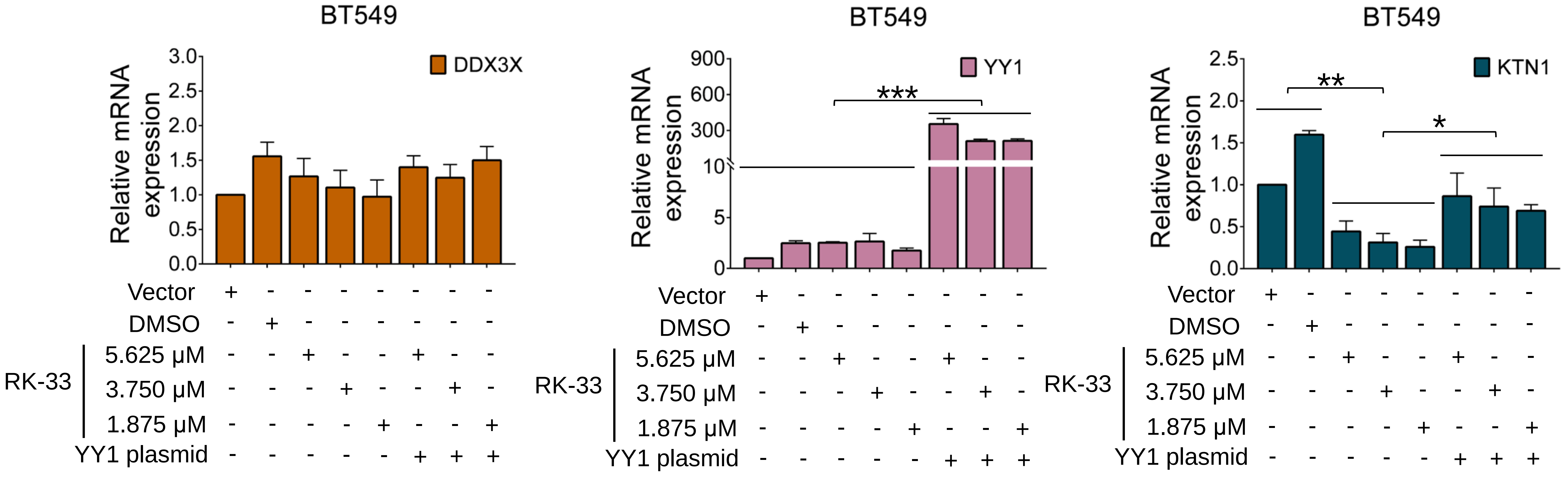

B

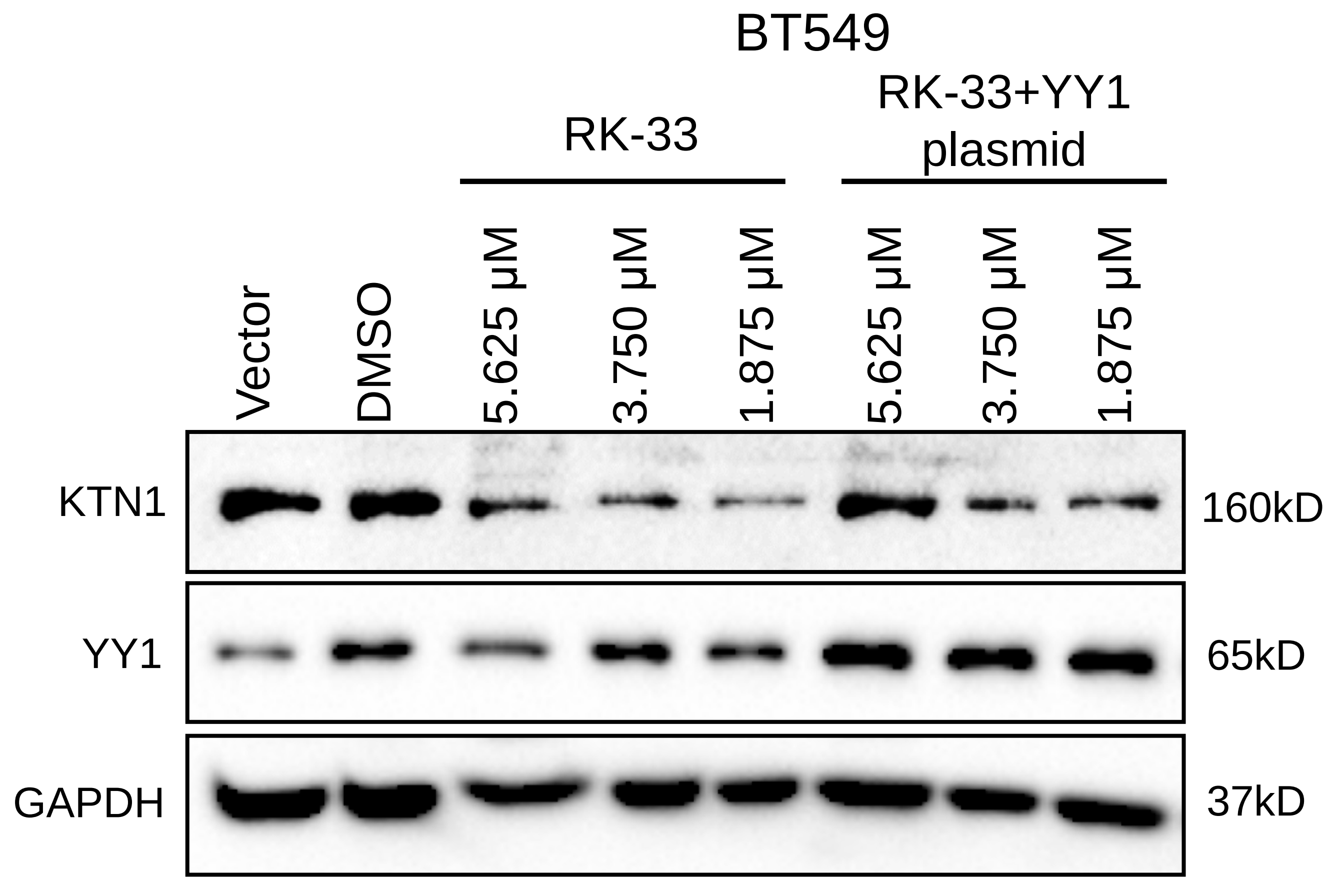

Supplementary Figure S7 Loss of DDX3X blocked the expression of KTN1 with DDX3X inhibitor treatment. (A) QRT-PCR analysis of the mRNA expression levels DDX3X, YY1, and KTN1 in BT549 cells treated with RK-33 (5.625 μM, 3.750 μM, or 1.875 μM) and/or expressing vector of YY1 for 48h. **(B)** Western blot analysis of the protein expression levels DDX3X, YY1, and KTN1 in BT549 cells treated with RK-33 (5.625 μM, 3.750 μM, or 1.875 μM) and/or expressing vector of YY1 for 48h. Error bars was shown by s.d., n≥3. * *P* < 0.05, ** *P* < 0.01 and *** *P*< 0.001 were showed compared with the negative control groups. The two-tailed t-test or ANOVA was used to assess *P*-values.

Supplementary Figure. S8

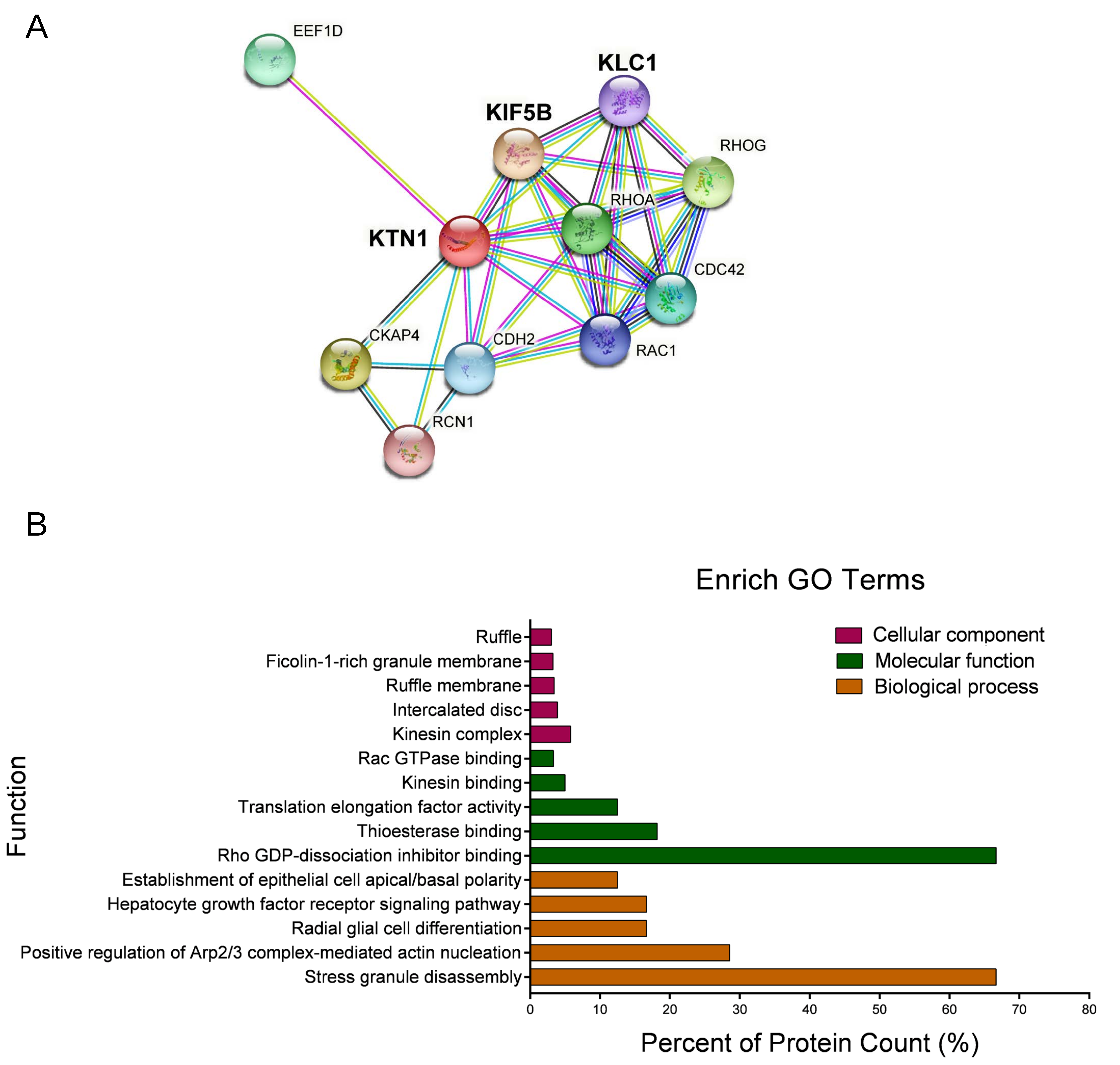

Supplementary Figure S8 The proteins analysis of interacting with KTN1 through STRING database (https://string-db.org/). (A) The interactional proteins with KTN1. **(B)** The top five Gene Ontology (GO) analysis of functional enrichments of KTN1-interacted proteins.

Supplementary Figure. S9

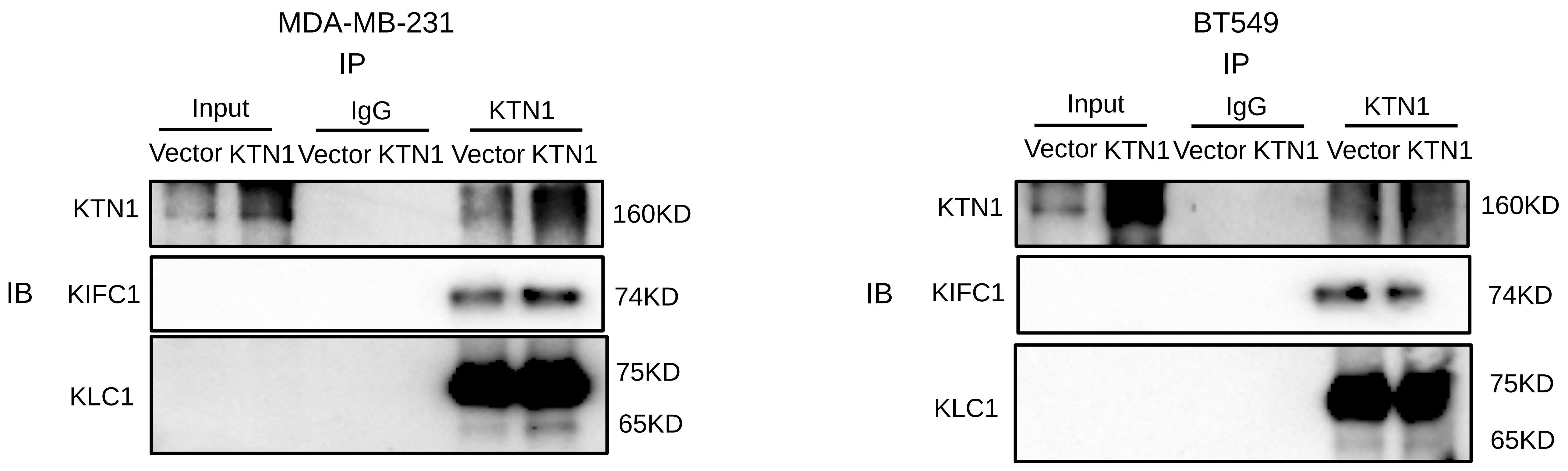

Supplementary Figure S9 Western blot and co-immunoprecipitation assays analysis of co-factor with KTN1 protein.
